# Targeting angiopoietin like-2 positive senescent cells improves cognitive impairment in adult male but not female atherosclerotic LDLr^−/−^;hApoB_100_^+/+^ mice

**DOI:** 10.1007/s11357-025-01763-x

**Published:** 2025-06-30

**Authors:** Mélanie Lambert, Géraldine Miquel, Gael Cagnone, Pauline Mury, Louis Villeneuve, Frédéric Lesage, Nathalie Thorin-Trescases, Eric Thorin

**Affiliations:** 1 Faculty of Medicine, Department of Pharmacology and Physiology, University of Montreal, Montreal, QC Canada; 2 Montreal Heart Institute, 5000 Belanger Street, Montreal, QC H1T 1C8 Canada; 3 University Hospital Sainte-Justine Research Center, University of Montreal, Montreal, QC Canada; 4 Ecole Polytechnique de Montréal, University of Montreal, Montreal, QC Canada; 5 Faculty of Medicine, Department of Surgery, University of Montreal, Montreal, QC Canada

**Keywords:** Cellular senescence, Cognition, AAV1-delivery of shRNA, *Inflammaging*, Morris water maze, SnRNA-seq

## Abstract

Cellular senescence contributes to cognitive decline in brain diseases. In dyslipidemic and atherosclerotic LDLr^−/−^;hApoB100^+/+^ (ATX) mice—a model exhibiting vascular dysfunctions and cognitive impairment—the role of senescence was investigated by targeting angiopoietin-like 2 (*angptl2*), a senescence marker. Adult ATX mice of both sexes received AAV1-sh-*angptl2*; cognition was assessed via the Morris Water Maze, cerebrovascular functions were evaluated in vitro and in vivo and hippocampal transcriptomic signatures were analyzed using single-nuclei RNA-sequencing. Sh-*angptl2* restored delayed memory retention in male mice, but impaired learning and short-term memory in females. Sh-*angptl2* had no effect on cerebrovascular functions. Transcriptomic analyses revealed sex-specific responses: endothelial senescence pathways were predominantly activated in male cells and repressed by sh-*angptl2*. In contrast, sh-*angptl2* up-regulated senescence pathways in all cell types in females. Key impacted senescent cell types were neurons, choroid plexus epithelial (CP-epithelial) cells, and oligodendrocyte precursor cells (OPCs), not endothelial cells. In male neurons, CP-epithelial cells and OPCs, gene-related pathways induced by sh-*angptl2* revealed enhanced neuronal energy metabolism but reduced synaptic pathways, higher synaptic formation with reduced intracellular signaling, structural remodeling and reduced synaptic development. In female cells, sh-*angptl2* disrupted GABAergic signaling despite promoting neuronal growth, up-regulated stress-response and decreased gene and protein processing, leading to inefficient neurogenesis. Although neuroprotective ApoE was upregulated in both sexes, males had higher expression of ApoE receptor subtypes, supporting better synaptic resilience. In conclusion, accumulation of non-vascular senescent cells contributes to cognitive impairment in male ATX mice, not females, highlighting the need for sex-specific senotherapies strategies in cardiovascular diseases.

**Supplementary Information:**

The online version contains supplementary material available at 10.1007/s11357-025-01763-x.

## Introduction

Research has established that age-related accumulation of senescent cells could cause chronic low grade inflammation, also known as *inflammaging* [1], through the release of factors from the Senescence-Associated Secretory Phenotype (SASP) [2]. Since the SASP includes a large range of proteases, chemokines, growth and pro-inflammatory factors with important paracrine and autocrine effects on cell and tissue biology, the *inflammaging* is believed to contribute, if not initiate, cardiovascular diseases (CVD) [3]. Senescent endothelial cells (EC) have been detected in cerebral microvessels [4] and other cell populations of the brain of aging mice [5], and altered functions of senescent cerebral cells may contribute to neurologic diseases and cognitive decline [6]. Indeed, different approaches to clear senescent cells have been able to improve cognition in mice with Tau-associated neuro-degeneration [7, 8], age-related cognitive impairments [9–11] and to improve gait function in hypertensive mice with cerebral microbleeds [12]. Altogether, these studies support a role for senescent vascular cells in vascular cognitive impairment (VCI) [13]. Accordingly, in a mouse model of atherosclerosis and VCI, characterized by micro-bleedings, blood brain barrier leakage, brain hypoperfusion and endothelial dysfunction [14], we recently showed that treatment with the senolytic ABT-263 (navitoclax) improved cognitive functions in male mice [15], although the cellular (vascular or non-vascular) origin of senescence was not evaluated.

Senolytics have entered clinical testing to treat age-associated brain diseases in both Alzheimer’s disease patients (SToMP-AD, NCT04685590) and patients with mild cognitive impairment (ALSENLITE, NCT04785300) at risk to develop Alzheimer’s disease [16]. These trials do not consider sex as a variable, but recent preclinical data showed that while male mice responded favorably to senotherapies, adverse cognitive effects were observed in healthy aging female mice treated with fesitin or dasatinib + quercetin [17] and in female mice with repeated mild traumatic brain injuries treated with navitoclax [18]. We also reported that navitoclax slowed learning process in middle-aged female atherosclerotic mice, while navitoclax improved cognitive functions in male mice [15]. Therefore, senotherapies as a new class of drugs may be ineffective or even deleterious for the female sex with CVD. In the present study, we used a unique senolytic approach that targets angiopoietin like-2 (*angptl2*) in our mouse model of VCI [14]. Angptl2 is a multifaceted circulating protein, with physiological roles in angiogenesis and development [19], but also with pathological inflammatory, oxidant and senescent properties in arteries [20]. Angptl2 is released by the SASP from EC, but also fibroblasts and other cell types such as microglia and oligodendrocytes and is considered a marker of senescence [21]. Recently, single-cell RNA sequencing of aged mouse brains listed *angptl2* as one of several senescence effector genes in cerebral microvascular EC [4]. Recombinant human ANGPTL2 promotes atherosclerosis in mice [22], while a single delivery of a shRNA targeting *angptl2* in young atherosclerotic mice slows aortic lesion growth by inducing apoptosis of senescent EC [23]. Some studies suggest that, through inflammatory pathways, angptl2 could exacerbate brain damage after ischemia–reperfusion [24] and induce neurodegeneration and cognitive decline [25]. Conversely, neuroinflammation and cognitive dysfunction induced by isoflurane anesthesia were attenuated in mice injected with shRNA targeting *angptl2* directly into cerebral ventricles [26]. Because angptl2 induces both inflammation and cellular senescence, and because inflammation and senescence seem to play a synergistic role in neurodegenerative diseases [27], our aim was to test whether targeting vascular *angptl2*^+^ senescent cells would delay the cognitive decline in severely dyslipidemic and spontaneously atherosclerotic LDLR^−/−^;hApoB100^+/+^ (ATX) mice. Since we previously reported that ATX mice exhibited sex-dependent responses to navitoclax [15], we also tested how male and female ATX mice responded to sh-*angptl2* in terms of cognition and transcriptomic signature.

## Methods

### Animals

Male and female ATX mice from our colony [14, 15] were randomly treated from 6 to 9 months old: they were intravenously injected with a bolus of 10^11^ of type 1 adeno-associated virus (AAV1) particles containing either a control shRNA scramble (sh-SCR) or a shRNA targeting *angptl2* mRNA (sh-*angptl2*), which was administrated twice at both 6 months and 7.5 months of age. Mice were sacrificed at 9 months of age, with a treatment duration of 3 months. All experiments were performed in accordance with the *Guide for the Care and Use of Experimental Animals of the Canadian Council on Animal Care* and the *Guide for the Care and Use of Laboratory Animals* of the US National Institutes of Health (NIH Publication No. 85–23, revised 1996). The study was approved by the Montreal Heart Institute Ethics Committee (ET No 2019–6203). Mice were kept under standard conditions (24 °C; 12 h:12 h light/dark cycle) and were fed ad libitum with regular chow (#2019S; Harlan Laboratories, Indianapolis, IN, USA).

### AAV1 production

The protocol was adapted from a previous study [23]. HEKT293T competent cells were plated until a confluence of 70% and were then transfected overnight with 12 µg/mL of the pXYZ C1 plasmid vector (serotype 1), 4 µg/mL of the plasmid containing sh-*angptl2* or sh-SCR (see shRNA sequences Table S1) and 48 µg/mL of polyethyleneimine (#764965, Sigma-Aldrich, St. Louis, MO, USA) in a starvation medium. The next day, the medium was changed, and cells were incubated for 48 h with normal growth medium. Then, cells were collected and lysed with Tris 1 M/NaCl 5 M pH 8.5. Lysis was accelerated by several freeze/thaw steps, and 1 µL of MgCl_2_ (1 M) in addition to benzonase (250 U/µL, #E1014, Sigma-Aldrich, St. Louis, MO, USA) was added *per* mL of lysate. After centrifugation, AAV1 were isolated from the supernatant by iodixanol gradients (#D1556, Sigma-Aldrich, St. Louis, MO, USA) during ultracentrifugation, then extracted with a syringe and concentrated in a phosphate-buffered saline with Tween 20 volume between 0.25 and 0.5 mL with Ultracel-100 regenerated cellulose membrane (100 kDa, #UFC910024, Millipore, St. Louis, MO, USA).

### AAV1 titration and administration in ATX mice

Titration was performed by qPCR reactions using a StepOnePlus Real-Time PCR System (Thermo Fisher Scientific, Waltham, MA, USA). AAV1 were quantified using a standard range made by serial dilutions (10 ng to 10^−6^ ng) of sh-*angptl2* and sh-SCR plasmids containing a target sequence (BGH). The primers for BGH target sequence were designed using Clone Manager software (Table S2).

### Morris water maze (MWM) test

MWM assesses learning and spatial memory [14, 15]. The water-maze apparatus consists of a white circular pool of 150 cm in diameter and 60 cm in height, filled with water made opaque with non-toxic white paint kept at a temperature of 22 °C. A plastic transparent platform (10 cm in diameter) was placed 1.5 cm below the water surface and 40 cm from the edge of the pool, except on day 1 (habituation phase) where the platform was visible and placed 0.5 cm above the water surface. The entire procedure took 11 days.

Mice were individually transferred from the home cage to the pool. Release points were balanced across four symmetrical positions on the pool perimeter. Each day of the test, mice underwent four trials during which they were allowed to freely swim for 60 s or until they found and climbed onto the platform; each trial was spaced from the other by a 30 min inter-trial interval. Platform finding was defined as staying on the platform for at least 3 s.

On day 1, during the habituation phase, mice that did not find the platform were trained in locating it by gently placing them on the platform for 30 s at the end of the trial. Then, 48 h later, the acquisition phase started and latency time to reach the hidden platform was measured (repeated for 5 days, from D1 to D5, in a row); mice that did not find the platform were trained in locating it by placing them on the platform for 30 s at the end of the trial.

On the fifth day of the acquisition phase (D5), 1 and 72 h after the last acquisition trial, the platform was removed from the pool, and each mouse was tested for short-term (1 h) and delayed (72 h) memory retention in a 60-s probe trial. During the probe trials, the time spent in the target quadrant (TQ, where the platform was located) versus the opposite quadrant (OQ) of the maze was scored as a reliable measure of short and delayed memory retention. The swim path of the mice and the time spent in the target quadrant were recorded by means of a computer-based video-tracking system (Smart version 3.0, Panlab/Harvard Apparatus, Holliston, MA, USA). All recordings were automatically quantified, without human intervention. Mice performed the MWM test twice, before the senolytic intervention (at the age of 6 months) and 3 months later (at the age of 9 months). Experimenters were blinded to groups during data acquisition.

### *In vivo *cerebral blood flow index

The day of the sacrifice, sh-SCR and sh-*angptl2* treated mice were anesthetized (initiation 5% isoflurane in O_2_ followed by maintenance at 1.5–2.5% isoflurane in 30% O_2_) and maintained at 37 °C, the scalp was excised and the bregma was positioned at the center of the images, targeting three different regions of interest: the retrosplenial cortex (RS), the primary motor cortex (M1) and the primary visual cortex (V1), in the left and the right brain. A bolus of indocyanine green (ICG, #1340009, Sigma-Aldrich, St. Louis, MO, USA) was intravenously injected in the tail vein, and time-series fluorescence imaging of ICG were generated, as described [28]. Six regions of interest (RS, V1 and M1, left and right brain) were scanned for fluorescence acquisition. Time-series fluorescence within the six regions were averaged. From the time-series images, the arrival time and the first peak time were recorded; from these parameters, a blood flow index (BFI) was calculated, representing the slope of the first fluorescent signal over time. BFI represents overall blood volume information with respect to time. Experimenters were blinded to groups during data acquisition.

### Atherosclerotic lesion quantification

Freshly isolated thoracic aortas of ATX mice were longitudinally opened. Because EC were scraped from freshly opened aortas for endothelial mRNA extraction [23], oil-red-O staining to visualize atherosclerotic plaque was not performed. Instead, after EC removal the aortas were rinsed with a saline solution to eliminate blood, fixed with 4% paraformaldehyde at 4 °C for at least 24 h, and white atherosclerotic lesions were photographed (with a Leica S8APO Zoom) and quantified. Atherosclerosis lesions were quantified by measuring areas of the lesions using ImageJ software. Plaque areas were expressed as percentage of total aortic area. Experimenters were blinded to groups during data acquisition.

### Cerebrovascular endothelium-dependent dilatory function

Mice were euthanized by terminal anesthesia (3.5% isoflurane in O_2_) followed by exsanguination. Then, mice were decapitated and brains rapidly removed and placed in ice-cold physiological salt solution (PSS; mmol/L: 130 NaCl; 4.7 KCl; 1.18 KH_2_PO_4_; 1.17 MgSO_4_; 14.9 NaHCO_3_; 1.6 CaCl_2_; 0.023 EDTA; 10 glucose; pH 7.4) aerated with 12% O_2_, 5% CO_2_ and 83% N_2_ at 37 °C. The middle cerebral artery was isolated, transferred to the arteriograph chamber (Living System Instrumentation, St Albans, VT, USA), cannulated and pressurized at 60 mm Hg for endothelial function assessment as previously described [15]. The artery segment was equilibrated and then sub-maximally pre-constricted with phenylephrine (1–3 µmol/L, #P1240000, Sigma-Aldrich, St. Louis, MO, USA); then dilatory responses were tested with a single cumulative exposure of incremental shear-stresses (0–20 dyn/cm^2^) induced by flow. Responses to flow were calculated as changes in vessel diameter from pre-constriction tone and expressed as % of maximal diameter. Experimenters were blinded to groups during data acquisition.

### Compliance of carotids

Compliance of carotids was also measured as previously described [29]. Passive pressure-diameter curves were conducted in carotid arteries in a Ca^2+^-free PSS containing 1 mM of EGTA to assess the mechanical properties of the arteries. Internal and external diameter changes were measured after each increment of intraluminal pressure (from 60 to 180 mm Hg with 20-mm Hg steps), to calculate mechanical parameters. The circumferential wall strain (Strain, %) was calculated according to [(*D* − *D*_initial mm Hg_)/*D*_initial mm Hg_], where *D* is the internal diameter at a given pressure and *D*_initial mm Hg_ is the initial diameter at the initial pressure (60 mm Hg). Experimenters were blinded to groups during data acquisition.

### Blood biochemistry

To determine whether chronic treatment with the AAV1 virus induces, or not, any toxicity, blood markers of liver function (glutamic-oxaloacetic transaminase (AST) and alkaline phosphatase) and renal function (plasma creatinine and urea) were measured in mouse plasma. Lipids (total cholesterol, LDL-cholesterol), triglycerides and glucose levels were also assessed. All analyses were performed in the laboratory of clinical biochemistry at the Montreal Heart Institute.

### Immunofluorescence staining [14, 15]

The day of the sacrifice, *n* = 3 mice *per* group were anesthetized and then perfused in the heart for 15 min with phosphate buffer saline (PBS), followed by 15 min with paraformaldhyde 4%, for a total of 30 min at 3 mL/min. Brains were removed, the hippocampus dissected and put at 4 °C for 24 h in 4% paraformaldhyde, prior to 24 h in 30% sucrose. They were then frozen in cold 2-methylbutane for 1 min and stored at -80 °C. Approximately three to four 20 µm slices of hippocampus were cut from hippocampus, in the dentate gyrus region, with a cryostat and put on Superfrost slides. Slides were then fixed for 1 h with paraformaldehyde 4%, permeabilized 20 min with Triton 0.5%, blocked 1 h with 2% bovine serum albumin (BSA), incubated 48 h at 4 °C with the primary antibody goat anti-angptl2 (12.5 µg/mL, #PA5-47139, Thermo Fisher Scientific, Waltham, MA, USA) diluted in 1% BSA and finally incubated 1 h at room temperature with the secondary antibody donkey anti-goat Alexa Fluor 555 (4 µg/mL, #A21432, Thermo Fisher Scientific, Waltham, MA, USA) diluted in 1% BSA and DAPI (1:1000). Each step was inter-stepped with three 5 min washes with PBS. Images were acquired with a LSM 710 confocal microscope (Zeiss, Oberkochen, Germany) using Plan Apochromat 40X/1.3 Oil DIC M27; images are maximum intensity projections created with Z-stack (0.5 µm Z-steps). Confocal images were analyzed using Image J to delimitate the area of positive cells, divided by the area of DAPI-labeled nuclei (% of total area). In each mouse, three brain slices within the hippocampus region were used for angptl2-immunofluorescence, and in each slice, two different images were taken; data presented are numbers of images. Since *n* = 3 mice *per* group were used, a total *n* = 18 images were quantified in each group. Experimenters were blinded to groups during data acquisition.

### Gene expression

RT-qPCR analyses were performed as previously described in the hippocampus [14, 15]. Total hippocampal RNA, or total RNA from native aortic EC scraped from fresh aortas [23], was reverse transcribed into first-strand complementary DNA with M-MLV reverse transcriptase (#28025–021, Thermo Fisher Scientific, Waltham, MA, USA), using random hexamer primers. The qPCR reactions were carried out on diluted RT products by using the DNA-binding dye SYBR Green PCR Master Mix (#4309155, Applied Biosystem, Waltham, MA, USA) to detect PCR products with BioRad CFX Real-Time PCR System. All samples were run in duplicate, and the fold changes in gene expression were calculated by a ΔΔCT method using the geometric mean of Cyclophilin A (*CycloA*), Hypoxanthine–guanine phospho ribosyl transferase (*HPRT*) and Beta-2-microglobulin (*BM2)* as housekeeping genes. The primers of target genes are listed in Table S3. To compare data from different qPCR plates, a common calibrator made of pooled SCR-males or pooled SCR-females was included in each plate. In each group, six mice were randomly selected for quantification.

### Single-nucleus suspension preparation

To evaluate the transcriptomic signature of the hippocampus, we used a snRNA-seq approach as previously described [30]. Special attention was used to obtain sufficient RNA quality (i.e. RNA integrity number > 6.5) that was achieved by dissecting out the hippocampus from mice brains and snap freezing them within 30 min of harvest. All the samples were stored at -80 °C and processed at the same time.

Fifty milligrams of the hippocampus was used to obtain single nuclei. They were isolated by combining enzymatic treatment with gentle mechanical dissociation using GentleMACS Octo Dissociator (Miltenyi Biotec, Gaithersburg, MD, USA). Briefly, frozen segments of hippocampus were put into the Nuclei Extraction Buffer (#130–128-024, Miltenyi Biotec, Gaithersburg, MD, USA) in C-Tube and processed for 5 min. After two steps of washing and filtration (100 and 70 µm + 20 µm stack together, respectively), nuclei were counted both automatically (CountessTM II Automated Cell Counter) and manually (hemacytometer). An average of 4,500,000 viable nuclei were extracted from each mouse hippocampus and were resuspended in 3–5 mL of buffer. Then, dilutions were made to load 10,000 nuclei at 700–1200 nuclei/µL in a Chromium Controller instrument (10X Genomics, Pleasanton, CA, USA).

Single-nucleus libraries were then prepared with the Next GEM Single Cell 3′ Dual Indexing kits (#PN-1000325, 10X Genomics, Pleasanton, CA, USA) using a Chromium Controller instrument (10X Genomics, Pleasanton, CA, USA) to generate single-nucleus gel bead-in-emulsion (GEMs). Then, GEMs were reverse transcribed into cDNAs that were amplified and cleaned up using SPRIselect Reagent Kit (#B23317, Beckman Coulter, Brea, CA, USA). The barcoded sequencing libraries were constructed by enzymatic fragmentation, end-repair, A-tailing, adaptor ligation, ligation cleanup, sample index PCR and PCR cleanup using 10X Genomics kit for sequencing in Illumina NovaSeq 6000 S4 PE100 platform.

### Single-nuclei RNAseq analysis

For 10X sequencing data processing, unique molecular identifier (UMI) counts were ranged into single digital gene expression matrices using 10X Genomics Cell Ranger package (V7.0.1, reference mm10-2020-A), resulting in an average *per* sample of ~ 5000 estimated number of nuclei, ~ 54,000 mean reads *per* nucleus, ~ 1300 median genes *per* nucleus, ~ 2500 median UMI counts *per* nucleus and ~ 24,400 total genes detected. Further analysis was performed using the Seurat package (V5). Before normalization, count matrices were adjusted for ambient RNA using the computational tool SoupX. We filtered out nuclei expressing either less than 100 genes or more than 10,000 genes, nuclei containing more than 30,000 UMIs, and nuclei expressing more than 10% mitochondrial genes. Single-nuclei transcriptomes were normalized *per* nucleus using the Seurat SCTransform function with regression on percent of mitochondrial genes and total number of genes. Following normalization, principal component analysis (PCA) on the most variable genes in the differential gene expression matrix identified 20 significant principal components, which served as input for Uniform Manifold Approximation and Projection (UMAP). To determine putative cell types on the embedded map, we used a density clustering approach (Louvain) and computed average gene expression for each identified cluster based on Euclidean distances in the UMAP space. We then compared each of the different clusters to identify marker genes that were differentially expressed across clusters, allowing cell type annotation. Doublets were estimated using DoubletFinder V2 and removed from subsequent analysis. Nuclei were assigned cell cycle phase (G1, S, G2/M) based on the expression of cell cycle genes using Seurat CellCycleScoring function. Replicates were integrated together using the SCT-based integration function (CCAIntegration method) from Seurat V5, and cell type-specific transcriptomic differences between conditions were statistically compared using the non-parametric Wilcoxon rank sum test on normalized RNA counts. Seurat visualization tools included Feature Plot, Dot Plot and UMAP plot. Single-nuclei gene expression profiles from each separate cell type identified by snRNAseq were further processed for differential analysis using Augur, Gene Set Variation Analysis (GSVA) and the Connectome R packages based on SCTranformed counts. Pathway enrichment analysis of differentially expressed genes (DEGs) (FindMarker’s Wilcoxon rank sum test on SCT count) was done using EnrichR. Heatmaps and circosplots were visualized using the R package ggplot2 and gplots.

### Availability of data

All single-nuclei data for this study have been deposited in the US National Center for Biotechnology Information gene Expression Omnibus database under the number GSE278331.

### Statistical analysis

Data are expressed as means ± standard error of the mean (SEM) of *n* mice. Group sizes were determined according to our previous studies [14, 15, 23]. Groups comparisons were analyzed using two-way analysis of variance (ANOVA) to compare the effect of both sex and treatment on mice, with repeated measures when adequate; Sidak’s or Tukey’s multiple comparison tests were performed. Unpaired, or paired, *t* test was adequately used to compare two groups. Statistical analyses were performed with the software Prism (Prism 10.0, GraphPad, San Diego, CA, USA), except for transcriptomic analyses that were performed with R software (v 4.1.2, R Core Team, 2021).

## Results

### Treatment with sh-*angptl2* was well tolerated

As expected, body weight was smaller in female than in male mice, and it was not affected by the two injections of AAV1-sh-*angptl2* or AAV1-sh-SCR over the 3 months of treatment (Fig. S1). In addition, in both male and female ATX mice, the treatment had globally little effect on blood markers of liver and renal functions and did not alter lipid and glucose plasma levels (Table S4). Only urea levels tended (*p* = 0.057) to be decreased by sh-*angptl2* in male mice (Table S4). When compared to males, female-sh-SCR mice also exhibited lower levels of triglycerides (Table S4).

### Treatment with sh-*angptl2* improved cognition of male mice only

**Fig. 1:**
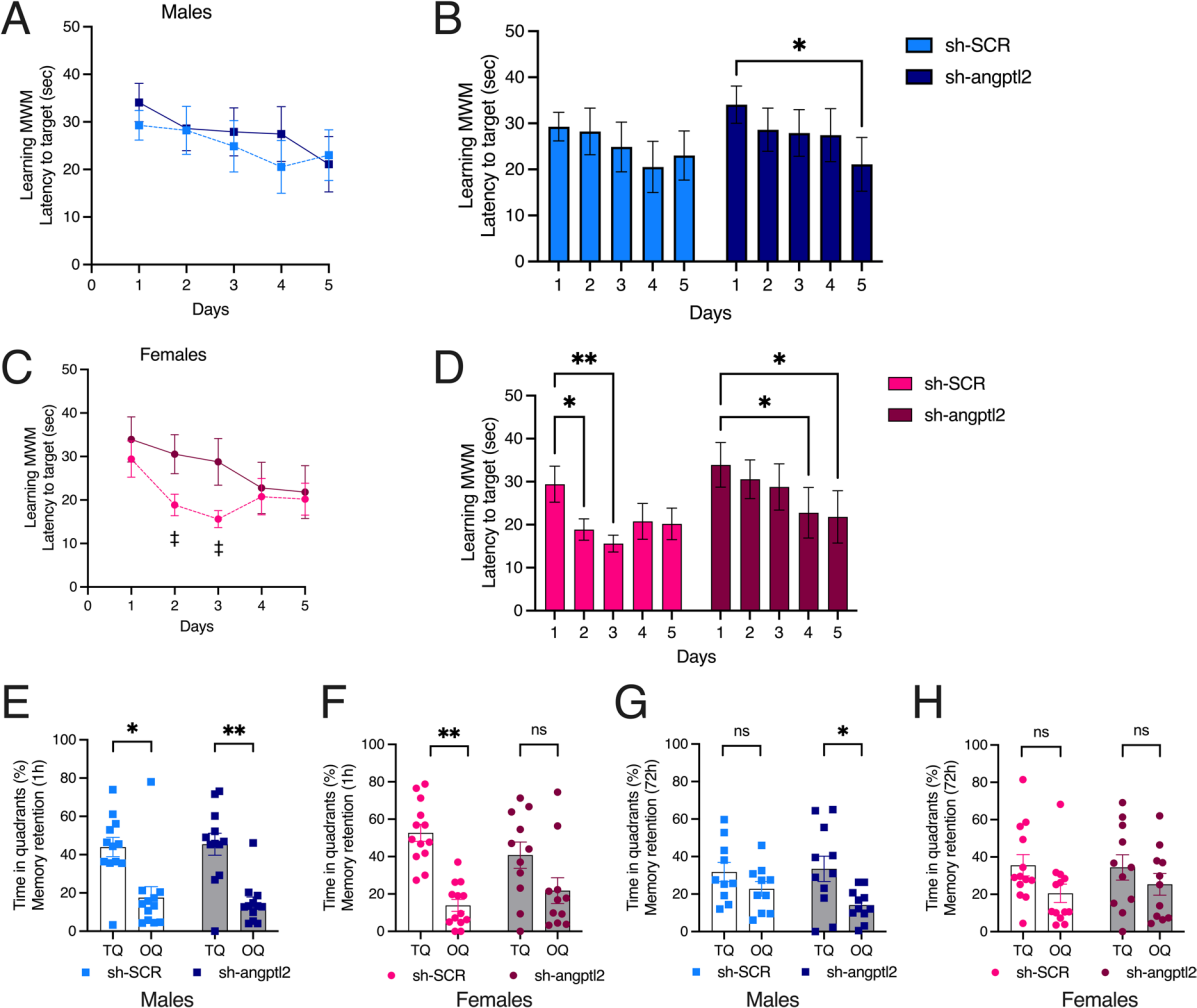
Impact of sh-*angptl2* treatment on cognition in the MWM test. **A** Latency to find the hidden platform from day 1 to day 5 in the acquisition phase (learning memory) of the MWM test in male (**A–B**) and female (**C–D**) ATX mice after treatment with sh-SCR or sh-*angptl2*. Each value is a mean of four trials performed by each mouse, *per* day of learning. ‡: *p* < 0.05 sh-SCR vs. sh-*angptl2,* in each day of learning, two-way ANOVA with repeated measures (Days × Treatment). Latency to target was also analyzed to determine in each group at what day the mice started to learn the localization of the hidden platform in male (**B**) and female (**D**) mice: * *p* < 0.05 vs. Day 1, two-way ANOVA with repeated measures (Days × Treatment) and Dunnet’s multiple comparisons test. (**E, F, G, H**) Percentage of time spent in the target quadrant (TQ, quadrant in which the platform was hidden) and in the opposite quadrant (OQ) by male and female mice after treatment with sh-SCR or sh-*angptl2* during the probe test. This probe test was performed on day 5, 1 h after the last acquisition of the learning phase (short-term memory retention, **E–F**) and 72 h after the end of the learning phase (delayed memory retention,** G–H**). *: *p* < 0.05 vs. TQ, two-way ANOVA with repeated measures (Sex × Treatment) and Sidak’s multiple comparisons test. *N* = 12 male mice (sh-SCR or sh-*angptl2*) and *n* = 13 female sh-SCR mice and *n* = 11 female sh-*angptl2* mice. Data are expressed as mean ± SEM of *n* mice. *: *p* < 0.05; **: *p* < 0.01

The MWM test was used to assess the impact of targeting senescent *angptl2*^+^ cells on learning and spatial memory [15]. Both male and female mice performed the MWM test prior to treatment (Fig. S2) and after 3 months of treatment with either sh-SCR or sh-*angptl2*, at 9 months of age (Fig. 1). As expected, 6-month-old mice of both sexes quickly learned to locate the immersed platform, and the latency time to reach the target decreased progressively over the 5 days (D1–D5) of memory acquisition (learning curve) (Fig. S2-AB). One hour after the last trial of the learning curve (on D5), the platform was removed, and mice were subjected to short-term memory retention test: the time spent in TQ (quadrant where the platform was previously located) and in OQ was measured (Fig. S2C). The same probe test was then repeated 72 h later, to assess delayed memory retention (Fig. S2D). The longer the mice stayed in the TQ, the better their memory retention. Short-term memory retention was similar in 6-month-old male and female mice (Fig. S2C); in both sexes, delayed memory retention was low and only significant in females (Fig. S2D). Therefore, before treatment, 6-month-old male and female ATX mice exhibited overall similar performance in the MWM test.

At 9 months of age, the impact of the treatment with either sh-SCR or sh-*angptl2* on cognition differed according to the sex of the mice (Fig. 1). In male ATX mice, learning was globally slow and similar after treatment with SCR or sh-*angptl2* (Fig. 1A), but with a significant shorter latency time to reach the immersed platform only observed on D5 of the learning phase in male sh-*angptl2* mice (Fig. 1B), suggesting a slight but statistically significant beneficial effect of the active treatment in male mice. In contrast, female ATX mice treated with sh-SCR learned faster than mice treated with sh-*angptl2*: on D2 and D3, the latency to reach the target was significantly lower in sh-SCR-females than in sh-*angptl2* mice (Fig. 1C) and while sh-SCR treated mice began to learn by D2, sh-*angptl2* mice learned on D4 and D5 (Fig. 1D), indicating a slower learning trajectory. This data suggests that targeting *angptl2*^+^ cells delays learning memory in female ATX mice.

Short-term memory retention was preserved in male ATX mice after sh-*angptl2* treatment (Fig. 1E). In contrast, while short-term memory retention was intact in sh-SCR-treated female mice, it was no longer present after treatment with sh-*angptl2* (Fig. 1F), suggesting again a deleterious cognitive effect of the active treatment in females. Remarkably, the sh-*angptl2* rescued delayed (72 h) memory retention in male ATX mice (Fig. 1G), but it had no beneficial effect in females (Fig. 1H). Therefore, these data show a sex-dependent cognitive effect of targeting *angptl2*^+^ cells, with a rescuing effect on retention memory in males and a deleterious effect on both learning and short-term memory retention in females.

### No effect of sh-*angptl2* on aortic, carotid or cerebrovascular function

**Fig. 2:**
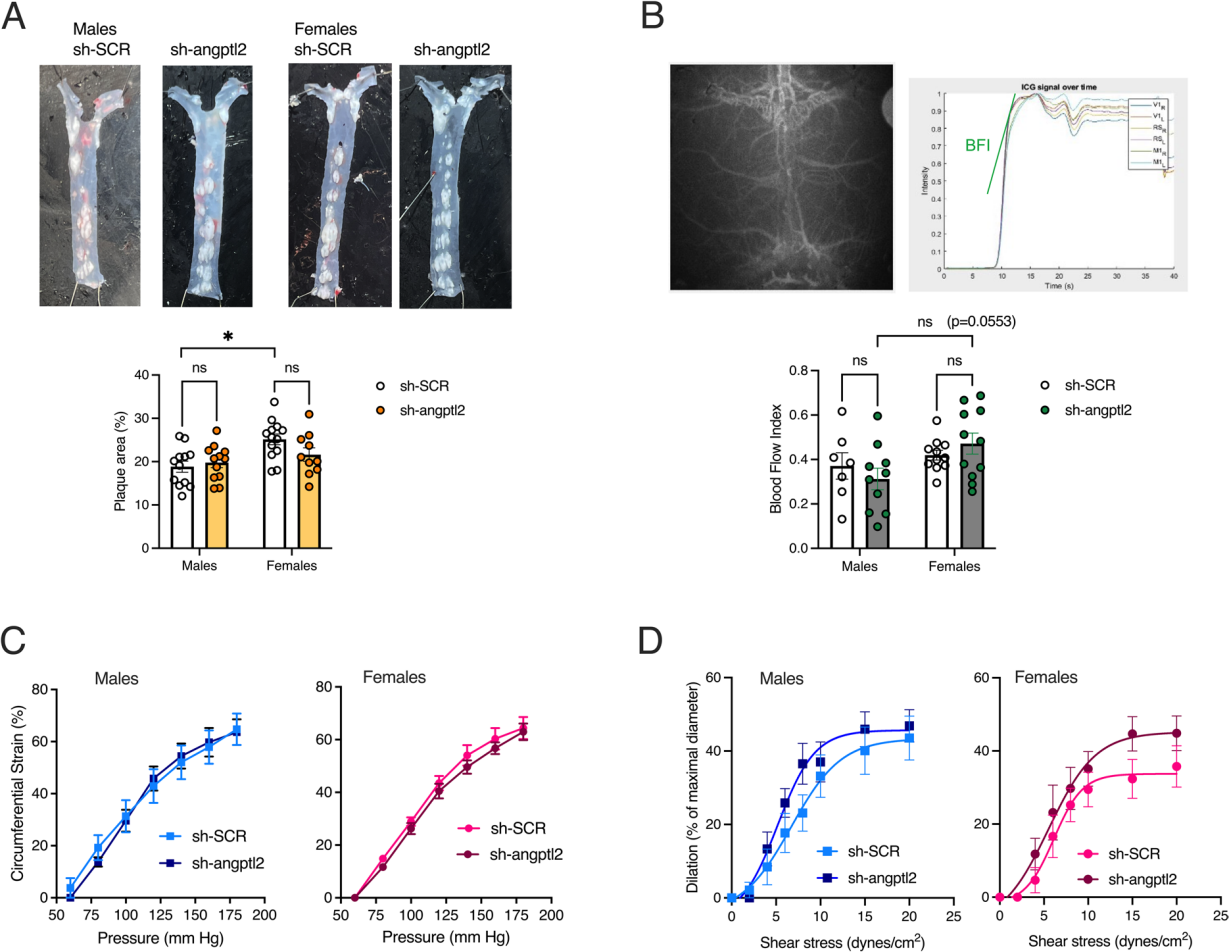
Impact of sh-*angptl2* treatment on atherosclerotic lesion, blood flow index, carotid compliance and cerebral endothelial dilatory function.** A** Typical images of thoracic aortas with atheroma plaques; lesions are expressed as percentage of total aortic area in both male and female ATX mice treated with sh-SCR or sh-*angptl2* (*n* = 12 males, *n* = 10–13 females). *: *p* < 0.05 vs. male-sh-SCR, two-way ANOVA (Treatment × Sex) and Tukey’s multiple comparisons test. **B** Time-series fluorescence imaging of ICG on a mouse head scalp and calculation of the cerebral BFI. A typical image of the fluorescent signal measured trough the open scalp of a mouse is presented. Six regions of interest (primary visual cortex (V1), retrosplenial cortex (RS) and primary motor cortex (M1), left (L) and right (R) brain) were scanned for fluorescence acquisition. Fluorescence was averaged within the six regions; the slope of the averaged time-series fluorescence signal corresponds to BFI. Two-way ANOVA (Treatment × Sex) and Tukey’s multiple comparisons test. **C** Carotid compliance of both male and female mice treated with sh-SCR or sh-*angptl2* (*n* = 8 males, *n* = 7–9 females), expressed as circumferential strain (%) in the presence of incremental changes in intraluminal pressure (mm Hg). **D** Flow-induced endothelium-dependent dilation of cerebral isolated pial arteries of both male and female mice treated with sh-SCR or sh-*angptl2* (*n* = 9 males, *n* = 8–10 females), expressed as dilation (% of maximal diameter) induced by cumulative increases in shear stresses (dynes/cm^2^). Data are mean ± SEM of *n* mice

The treatment did not modify the size of the aortic atherosclerotic plaques neither in male (18.8% ± 1.3 vs. 19.8 ± 1.2%, *n* = 12, sh-SCR vs. sh-*angptl2*) nor in female (25.2 ± 1.3% vs. 21.6 ± 1.6%, *n* = 13 and *n* = 10, sh-SCR vs. sh-*angptl2*) ATX mice (Fig. 2A). Of note, females-sh-SCR exhibited a larger aortic plaque than male-sh-SCR (*p* = 0.0054). ATX mice do not develop plaque in cerebral arteries.

In vivo brain blood flow was assessed by ICG-fluorescence imaging (Fig. 2B). BFI is the slope of the peak of ICG fluorescence in the intensity-time curve; BFI represents the overall blood volume with respect to time. The treatment with sh-*angptl2* did not affect BFI, but female mice tended (*p* = 0.0553) to exhibit a higher BFI than male ATX mice in the sh-*angptl2* group (Fig. 2B).

Finally, ex vivo*,* neither carotid elastic properties, i.e. carotid compliance (Fig. 2C), nor endothelium-dependent dilatory responses to shear stress of isolated pressurized cerebral pial arteries (Fig. 2D) were affected by targeting *angptl2*^+^ cells, in both male and female mice. Neither EC_50_ (the shear stress that induces 50% of dilation), nor E_max_ (the maximal endothelium-dependent dilation) was significantly different between mice treated with sh-SCR or sh-*angptl2*, in both sexes. Altogether these data show that targeting *angptl2*^+^ cells did not affect aortic, carotid or pial and cerebral micro-vascular functions in adult ATX mice, of both sexes.

### Impact of sh-*angptl2* treatment on angptl2 expression

**Fig. 3:**
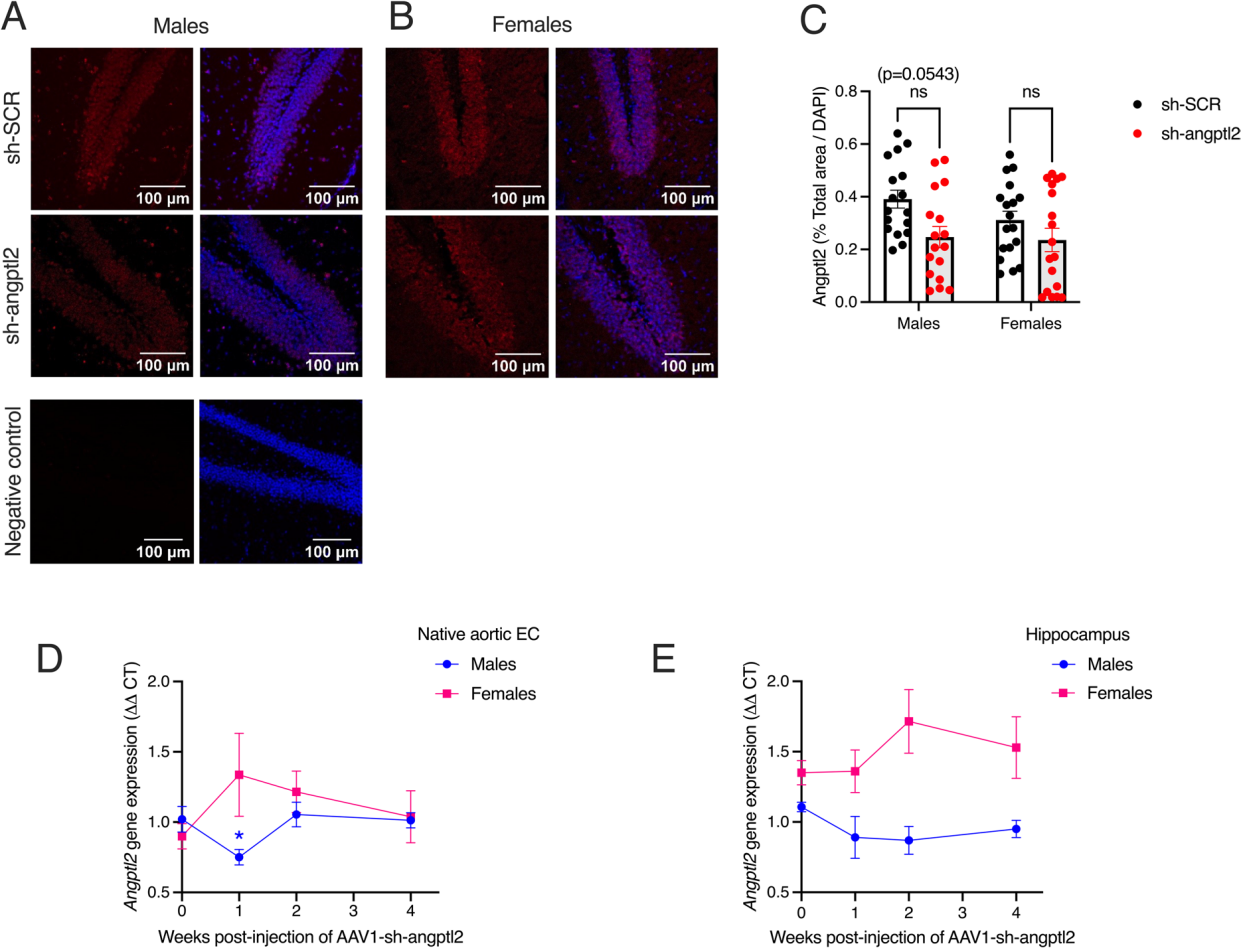
Impact of sh-*angptl2* treatment on angptl2 protein and gene expression. Typical images of confocal immunostaining for angptl2 (in red; nuclei stained in blue with DAPI) in hippocampal slices from male (**A**) and female (**B**) ATX mice treated with sh-SCR or sh-*angptl2*. All images were taken in the dentate gyrus of the hippocampus. **C** Average hippocampal expression of angptl2 in male and female ATX mice treated with sh-SCR or sh-*angptl2* analyzed using Image J. Two-way ANOVA (Sex × Treatment) and Tukey’s multiple comparisons test. Data are mean ± SEM of *n* = 18 images collected in three mice, *per* group. **D** *Angptl2* gene expression measured by RT-qPCR in native aortic EC scraped from the aorta the day of the sacrifice, in mice after 1, 2 or 4 weeks of treatment with sh-*angptl2*. *: *p* < 0.05: *vs.* untreated mice on week 0, one-way ANOVA and Dunnett’s multiple comparisons test. Data are expressed as mean ± SEM of *n* = 6 mice of both sexes. **E** *Angptl2* gene expression measured by RT-qPCR in the hippocampal region in mice after 1, 2 or 4 weeks of treatment with sh-*angptl2*. Data are expressed as mean ± SEM of *n* = 6 mice of both sexes

Angptl2 protein levels assessed by immunofluorescence in the dentate gyrus of the hippocampus tended (*p* = 0.054) to decrease in sh-*angptl2* treated male mice (from 0.39 ± 0.03 in sh-SCR to 0.25 ± 0.04% in sh-*angptl2*); in contrast, the treatment had no effect on females (*p* = 0.502) (Fig. 3A–C). The inhibitory effect of the sh-*angptl2* is relatively low as it decreased angptl2 protein expression by 37%, and this effect was limited to male mice. This could be due to the fact that angptl2 protein expression was measured 1.5 months after the second injection of sh-*angptl2*, a time during which angptl2 expression could have slowly returned to normal levels. Thus, to assess the early impact of sh-*angptl2*, *angptl2* mRNA levels were measured by RT-qPCR in native aortic EC scraped from fresh aortas and in the brain hippocampus region from small, independent groups of ATX mice treated with AVV1-sh-*angptl2* for 1, 2 or 4 weeks; we previously reported that sh-*angptl2* significatively inhibited *angptl2* gene expression in native EC from young male ATX mice [23]. These groups of mice were injected with one unique batch of AAV1 virus to limit variability in treatment efficacy and only used to measure *angptl2* mRNA expression. Compared to baseline (before treatment at *t* = 0), *angptl2* mRNA levels in native EC significantly decreased after 1 week in male ATX mice treated with sh-*angptl2* (from 1.02 ± 0.09 to 0.75 ± 0.06, *n* = 6, control vs. sh-*angptl2*, *p* < 0.05; Fig. 3D); after 2 or 4 weeks, however, *angptl2* levels returned to control levels (Fig. 3D). In females, sh-*angptl2* treatment did not lower *angptl2* mRNA levels in native EC (Fig. 3D). Similarly, sh-*angptl2* treatment tended to decrease *angptl2* mRNA levels in the hippocampus of male mice after 1 and 2 weeks of treatment, but not in females (Fig. 3E). Hence, these data suggest that the sh-*angptl2* transiently lowered *angptl2* mRNA and protein expression, at least in male mice. Of note, control sh-SCR was not tested, but we previously reported that treatment of ATX mice with sh-SCR had no impact on *angptl2* mRNA expression over time [23].

### Impact of sh-*angptl2* treatment on single nuclei mRNA transcripts of the mouse hippocampus

To comprehend at the cellular and molecular levels the sex differences observed after sh-*angptl2* treatment on cognition, snRNA-seq was performed on the hippocampal region of *n* = 3 mice in each group of male and female mice. Mice were selected based on their short (1 h) and delayed (72 h) memory retention to be representative of the whole population of mice studied: in these 12 selected mice, short-term memory retention in male mice was preserved with either treatment, whereas short-term memory retention was lost in females treated with the sh-*angptl2*, but not the sh-SCR (Fig. S3A). In addition, sh-*angptl2* treatment restored delayed memory retention in male mice, but not in female mice that lost delayed memory retention after the active treatment (Fig. S3B).

**Fig. 4:**
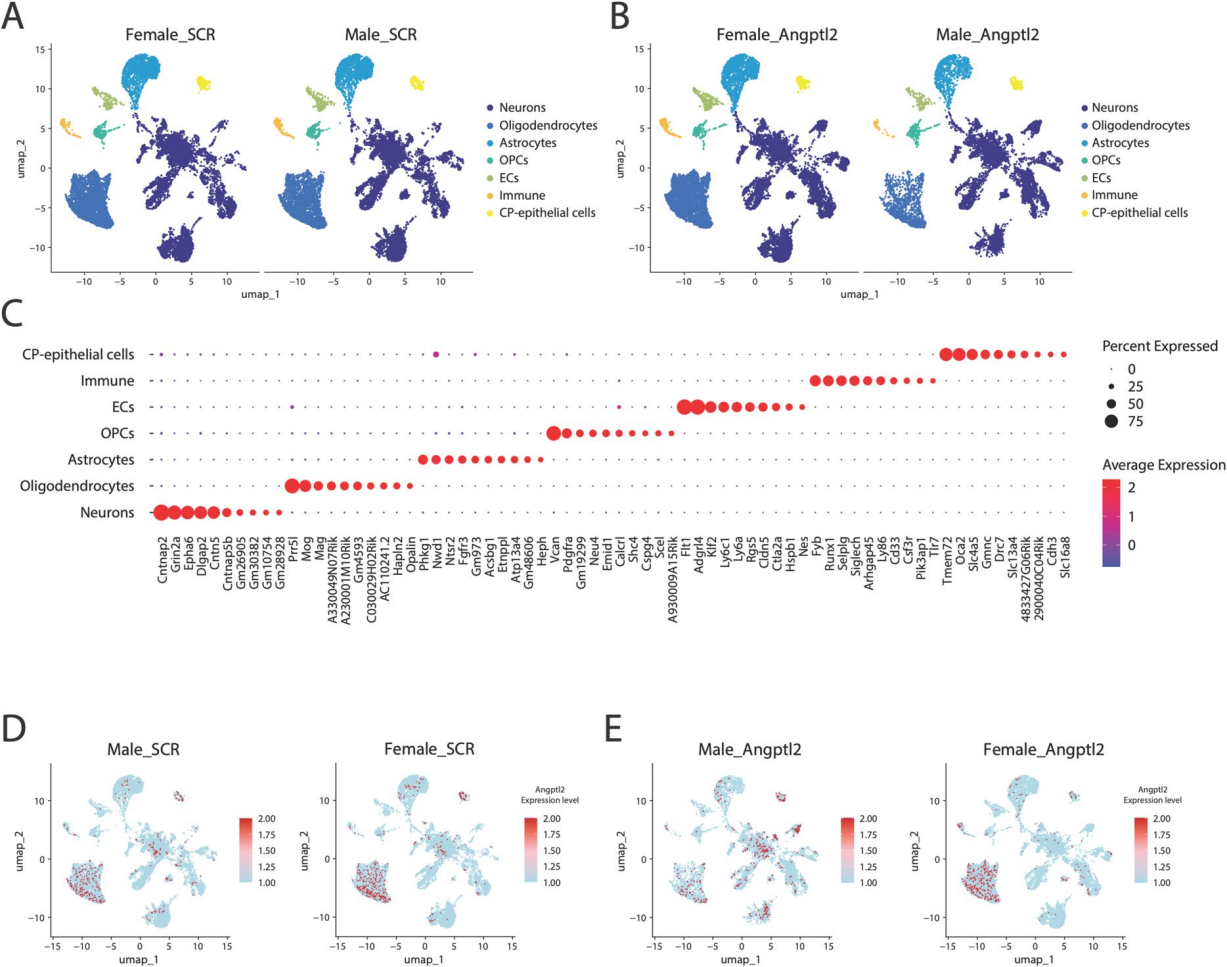
Impact of sex and sh-*angptl2* treatment on brain cell types from ATX mice. **A** Uniform manifold approximation and projection plot, in two dimensions (UMAP-1 and UMAP-2), of annotated cell-types clustered based on their specific gene expression, present in hippocampus from male and female ATX mice treated with sh-SCR (**A**) or sh-*angptl2* (**B**). **C** Dot plot showing the expression of the top 10 gene markers used to identify seven distinct cell clusters. In the plot, dot color represents average gene expression, with values scaled from -1.0 (blue) to 2.0 (red), dot size reflects the percentage of cells expressing each gene, ranging from 0% (smallest) to 75% (largest). **D–E** Feature UMAP plots showing the distribution of *angptl2* expression across the entire data set, stratified by sex (male or female mice) and treatment (sh-SCR in **D** or sh-*angptl2* in **E**); average expression of *angptl2* is indicated by color with values scaled from -1.0 (blue) to 2.0 (red)

We first clustered hippocampal nuclei using unsupervised graph-based clustering and visualized them as a UMAP graph to identify the cell types in both male and female mice treated with sh-SCR (Fig. 4A) or sh-*angptl2* (Fig. 4B). We identified seven cell family types: neurons (61% of total cells in average), oligodendrocytes (20%), astrocytes (11%), oligodendrocytes progenitor cells (OPCs; 2.9%), ECs (2.0%), immune cells (1.6%) and choroid plexus epithelial cells (CP-epithelial cells; 1.5%), using top 10 expressed gene markers (Fig. 4C). The proportion of cell types was globally conserved between the conditions, except for male neurons and male oligodendrocytes that increased and decreased in the sh-*angptl2* group, respectively (Table S5). Then, because the treatment targeted *angptl2* mRNA, *angptl2* gene expression within the seven cell clusters was identified (Fig. 4D–E).

### *Angptl2* transcripts are heterogeneously reduced in male cells only

**Fig. 5:**
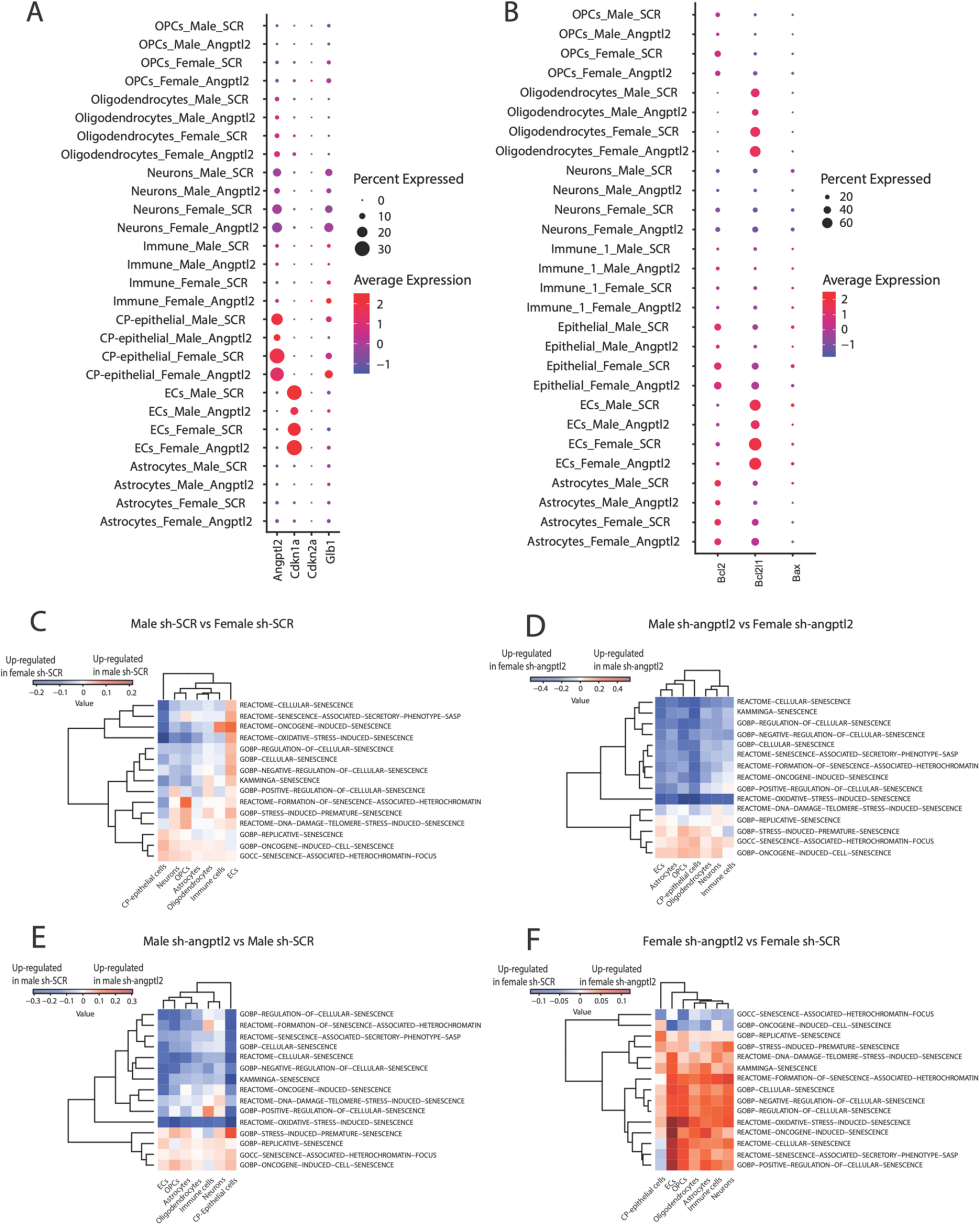
Impact of sex and sh-*angptl2* treatment on senescent pathways. Dotplot of senescent (**A**) and apoptotic (**B**) markers gene expression in hippocampal cell types across the four different conditions. Average expression of senescent and apoptotic markers is indicated by color with values scaled from -1.0 (blue) to 2.0 (red); the percentage of cells expressing the markers is indicated by dot size, scaling from 0 to 30% (for senescence) or from 20 to 60% (for apoptosis). **C–F** Heatmaps displaying GSVA scores for selected senescence-related gene sets across hippocampal cell types; comparisons are shown between male and female sh-SCR (**C**), male and female sh-*angptl2* (**D**), between male sh-SCR and male sh-*angptl2* (**E**) and between female sh-SCR and female sh-*angptl2* (**F**). Rows represent senescence gene-related pathways (log_2_ fold change expression), columns represent cell types. The color of each cell in the matrix represents the expression level of a specific pathway: red corresponds to higher expression, blue corresponds to lower expression, in male vs. female-sh-SCR, scaling from -0.2 to 0.2 (**C**), in male vs. female-sh-*angptl2*, scaling from -0.4 to 0.4 (**D**), in male-sh-*angptl2 vs.* male-sh-SCR, scaling from -0.3 to 0.3 (**E**), and in female-sh-*angptl2 vs.* female-sh-SCR, scaling from -0.1 to 0.1 (**F**). On the heat-maps, the left dendrograms show the hierarchical clustering of gene pathways based on the similarity of their expression profile

As observed in hippocampal slices by immunostaining, *angptl2* levels, as well as the number of cells expressing it, were significantly decreased in male mice treated with sh-*angptl2*, but not in females: *angptl2* levels were low and not modified in ECs, but lowered in CP-epithelial cells (-75%), neurons (-50%) and oligodendrocytes and OPCs (-10%), but only in male mice (Fig. 5A). In contrast, *angptl2* was minimally affected in female cells, with a small inhibition (-10%) in CP-epithelial cells, no effect in neurons, OPCs or immune cells, pointing to sex-specific and cell-dependent regulatory mechanisms of sh-*angptl2* (Fig. 5A). Importantly, regardless of treatment or sex, *angptl2*-positive cells overlap with senescent CP-epithelial cells, neurons, and to a lesser extent OPCs, all of which were positive for β-galactosidase (*glb1*); ECs, that express low levels of *angptl2,* were positive for the senescence marker p21 (*cdkn1a*) (Fig. 5A). The reason why senescent *angptl2*^+^ cells are closely associated with *glb1*, but not with *cdkn1a*, needs further studies since both are recognized markers of senescence.

### Impact of the sh-*angptl2* treatment on senescence and apoptotic markers

Next, using the snRNA-seq data, we validated that sh-*angptl2* reduced senescent load by promoting apoptosis of senescent cells, as it is expected from a senolytic approach. Transcripts of senescence markers p16 (*cdkn2a*), *cdkn1a* and *glb1* and of apoptotic markers bcl-2 (*bcl2)*, bcl-X/L1 (*bcl2L1*) and bax (*bax)* were measured in bulk hippocampus (Table 1) and *per* cell-type (Fig. 5A–B). In male hippocampal cells, when compared to sh-SCR, sh-*angptl2* strongly decreased *cdkn1a* transcripts and slightly increased *glb1*; the % of cells expressing both markers were low (Table 1), and *cdkn1a* was mostly expressed in EC (Fig. 5A). In contrast, in female brain cells, sh-*angptl2* increased both *cdkn1a* and *glb1* transcripts (Table 1); the % of cells expressing *cdkn1a* and *glb1* were five- and twofold higher in female than in male sh-*angptl2* treated cells, respectively (Table 1), and *cdkn1a* was mostly expressed in EC (Fig. 5A). The senescent marker *cdkn2a* was not detectable in the data set of both sexes (Table 1; Fig. 5A). In male cells, sh-*angptl2* strongly decreased anti-apoptotic *bcl2L1* and *bcl2* transcripts (Table 1), in various cell-types including EC, oligodendrocytes, CP-epithelial cells and neurons (Fig. 5B). Pro-apoptotic *bax* was expressed at lower levels and was also reduced by sh-*angptl2* in male cells (Table 1). In contrast, in female cells, neither *bax* nor *bcl2* transcripts were affected by sh-*angptl2,* only *bcl2L1* was slightly reduced (Table 1; Fig. 5B). The % of cells expressing *bcl2L1* and *bax* were twofold higher in female than in male sh-*angptl2*-treated cells (Table 1). Altogether, these data support the concept that in male ATX mice, sh-*angptl2* targets senescent *p21*^+^ EC cells, and senescent non-vascular cells, particularly *glb1*^+^ neurons and CP-epithelial cells, and eliminates them by inducing their apoptosis, lifting the anti-apoptotic effect of *bcl* genes. In females, in contrast, sh-*angptl2* promotes senescence, despite a small pro-apoptotic effect.

**Table 1:** Impact of sh*-angptl2* on changes in gene expression (snRNA-seq) of senescence and apoptotic markers in hippocampal cells (all cell-types combined) in male and female mice

Males	Avg log_2_ FC	*p* adj val	% sh-*angptl2*	% sh-SCR
*Cdkn1a*	− 1.131	4.28E − 15	0.007	0.02
*Cdkn2a*	− 4.443	1	0	0
*Glb1*	0.199	2.20E − 07	0.060	0.084
*Bcl2*	− 0.017	2.14E − 10	0.107	0.142
*Bcl2L1*	− 0.888	2.69E − 119	0.153	0.277
*Bax*	− 0.236	3.02E − 39	0.059	0.109
**Females**				
*Cdkn1a*	0.543	5.66E − 09	0.033	0.020
*Cdkn2a*	5.144	1	0.001	0
*Glb1*	0.160	2.13E − 10	0.111	0.084
*Bcl2*	− 0.271	1	0.187	0.182
*Bcl2L1*	− 0.005	4.65E − 10	0.394	0.334
*Bax*	0.017	0.002	0.112	0.092

*Cdkn1a*: cyclin-dependent kinase inhibitor 1a (p21); *Cdkn2a*: cyclin-dependent kinase inhibitor 2a (p16); *Glb1*: β-galctosidase-1; *Bcl2*: Bcl2 apoptosis regulator; *Bcl2L1*: Bcl-X; *Bax*: Bcl2 associated X, apoptosis regulator; % sh-*angptl2*: percentage of cells expressing the transcript in sh-*angptl2*; % sh-SCR: percentage of cells expressing the transcript in sh-SCR; avg log_2_FC: average log_2_ fold change in expression between sh-*angptl2* and sh-SCR condition; *p* adj val, *p* value adjusted after Bonferroni correction

### Impact of the sh-*angptl2* treatment on senescent pathways

Next, we used the GSVA method to evaluate pathway enrichment scores in each individual cell type of the hippocampus of mice in each group (Fig. 5C–F). Angptl2 being a marker of senescence, we focused on senescence hallmark pathways in hippocampal cells from male and female mice treated with sh-SCR or sh-*angptl2*. In mice treated with sh-SCR, the analysis revealed that EC had a totally different pattern when compared to the other cell types. Indeed, senescence-related pathways are overexpressed (red signals) in male EC when compared to female EC (Fig. 5C), confirming our results in EC from peripheral arteries of men and women CAD patients [30]. CP-epithelial cells, however, expressed more senescence pathways in female-SCR than male-SCR cells (Fig. 5C). In all the other cell types, senescence pathways are globally equally distributed between female (blue signals) and male cells (red signals) (Fig. 5C). Hence, the atherosclerotic phenotype is associated with senescence in male and female hippocampal cells.

Then, by comparing male and female cells treated with sh-*angptl2*, the presence of mostly bright blue signals (Fig. 5D) highlights senescence pathways in female cells, confirming that the treatment cleared senescent cells only in male hippocampal cells (Table 1). Thus, when we compared male cells treated with sh-SCR vs. sh-*angptl2* the senescent pathways are up-regulated in male mice treated with sh-SCR (blue signals) in all cell types (Fig. 5E), demonstrating the efficiency of sh-*angptl2* to clear senescent cells in the hippocampus of male mice. In contrast, in female cells, the presence of bright red signals illustrates up-regulation of senescence pathways by the active treatment (Fig. 5F). Of note, the amplitude of changes in senescence pathways is sex-dependent: the sh-*angptl2* treatment had twofold more impact on senescence pathways in males (values from ± 0.3) than in females (values from ± 0.15). Thus, the treatment with sh-*angptl2* had an opposite effect in male and female mice, an expected reduction in senescence in male cells, but a paradoxical (small) increase in senescence in female cells.

### Heterogenous cell-specific transcriptomic remodeling by senolysis

**Fig. 6:**
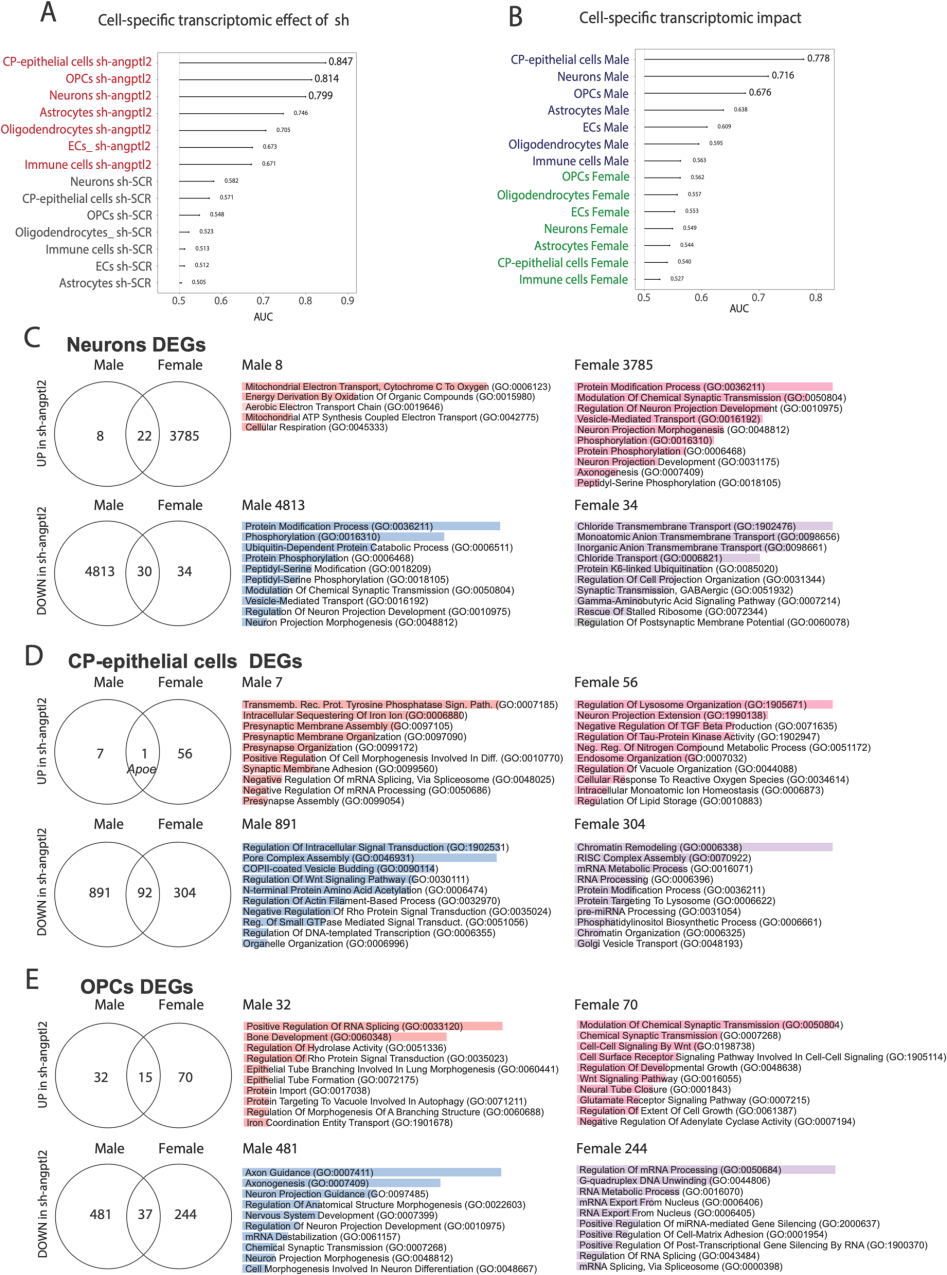
Differentially expressed genes in the three main cell types involved in the sex-specific response to sh-*angptl2* treatment in mice. Identification of the cell types most responsive to biological perturbations in the data set using Augur method: identification of cell-specific transcriptomic effect of the treatment (sh-*angptl2* and sh-SCR) (**A**) and of the sex (**B**) in hippocampal cell types. Augur provides an unbiased AUC score, which measures how well the model can separate two groups of cells based on their gene expression profiles: the higher the AUC (> 0.5 and the closest to 1.0) in a cell type, the most likely this cell type is involved in the biological response. **C**, **D**, **E** Venn diagrams showing the overlap of DEGs, both upregulated and downregulated, in male and female cells following sh-*angptl2* treatment. Associated gene pathways (Gene Ontology Biological Process; *p* < 0.05, log₂ fold change ≥ 0.25, minimum percent expression ≥ 10%) were identified using EnrichR for neurons (**C**), CP-epithelial cells (**D**) and OPCs (**E**). In the associated gene pathway analysis, bar length corresponds to the number of DEGs associated with each pathway, reflecting the strength of the gene set enrichment

Next, using Augur, a method that identifies the cell types most responsive to biological perturbations in the data set, we were able to identify the cell-specific transcriptomic effect of the treatment (Fig. 6A) and the impact of the treatment depending on the sex (Fig. 6B). Augur provides an unbiased area under the curve (AUC) score, which measures how well the model can separate two groups of cells based on their gene expression profiles: the higher the AUC (> 0.5 and the closest to 1.0) in a cell type, the most likely this cell type is involved in the biological response.

Augur analysis shows that CP-epithelial cells, OPCs and neurons were the three cell types that are the most responsive to the sh-*angptl2* with AUC ≥ 0.8, indicating that the variations in their gene pools were the highest in response to the treatment (Fig. 6A). EC also responded well as indicated by an AUC of 0.673 (Fig. 6A). As expected, all AUC in sh-SCR cells were < 0.6 (Fig. 6A) confirming the “placebo-like” effect of the AAV1 delivering a scramble sh-RNA. Importantly, AUC values are all higher in male than in female, for every cell type (Fig. 6B), demonstrating that the active treatment had higher effects in male cells, when compared to female cells. The sh-*angptl2* had a major impact on male CP-epithelial cells (AUC 0.778), neurons (AUC 0.716), and OPCs (AUC 0.676), and to a lesser extent in EC (AUC 0.609) (Fig. 6B). These results are consistent with the sustained reduction of *angptl2* expression observed specifically in male CP-epithelial cells, neurons and, to a lesser extent in OPCs, in the sh-*angptl2* group (Fig. 5A).

Thus, for the rest of the analysis, we focused on CP-epithelial cells, neurons and OPCs. Using EnrichR method, we performed a differentially expressed genes analysis: in each of the three cell types, we observed distinct patterns of DEGs and related pathways in response to sh-*angptl2* treatment.

### DEGs in neurons

We first focused our analysis on neurons since they are the cells ultimately responsible for cognitive function. The sh-*angptl2* had a sex-dependent opposite effect on the number of DEGs: in male neurons, only eight DEGs were up-regulated compared to 3785 DEGs in females. Conversely, 4813 DEGs were repressed in male neurons but only 34 in females (Fig. 6C). In male neurons, up-regulated pathways focus on mitochondrial function and neuronal energy metabolism: enhanced pathways related to electron transport and ATP synthesis suggest heightened metabolic demands (Fig. 6C); this, however, is only based on eight DEGs. Down-regulated pathways in male neurons mainly concern protein modification and synaptic processes: reduction in pathways related to protein phosphorylation, synaptic transmission modulation and vesicle-mediated transport suggests reorganization in synaptic communication between neurons, with reduced protein function and overall neuronal signaling (Fig. 6C). In female neurons, pathways analysis points to protein modifications, synaptic signaling and neuron projection development (Fig. 6C), suggesting reorganized growth and communication between neurons. Down-regulated pathways in female neurons highlight a dysfunction in the control of the excitability of neurons through a reduction in chloride and anion transport pathways and down-regulation of GABAergic signaling (Fig. 6C). Overall, these findings indicate that male neurons may experience increased energy availability but reduced support for synaptic function, while female neurons may thrive in growth and communication at the cost of excitatory-inhibitory balance.

### DEGs in CP-epithelial cells

These cells form a structural cellular lining of the choroid plexus; they secrete the cerebrospinal fluid (CSF) and various growth factors [31], and they form the blood-CSF barrier, suggesting a key role of CP-epithelial cells in cerebrovascular homeostasis. Like neurons, CP-epithelial cells responded to the treatment, which down-regulated more DEGs than it up-regulated DEGs, both in male CP-epithelial cells (891 down DEGs vs. seven up DEGs) and in female CP-epithelial cells (304 down DEGs vs. 56 up DEGs) (Fig. 6D). Only one gene was upregulated in both male and female CP-epithelial cells, *Apoe*, which encodes for ApoE, a protein that regulates lipid transport in the CSF and has neuroprotective anti-inflammatory properties [32]. In male CP-epithelial cells, up-regulated pathways concern presynaptic and synaptic morphogenesis, and gene metabolic processes, suggesting enhanced cell differentiation, synaptic formation and changes in cellular structures (Fig. 6D). Down-regulated pathways point to a reduction in intracellular signaling, protein transport, cytoskeletal organization and gene transcription regulation, which could result in reduced structural organization, and protein stability and trafficking (Fig. 6D). In female CP-epithelial cells, up-regulated pathways refer to cellular organization, metabolic regulation and stress response (Fig. 6D). This indicates increased cellular processes for maintaining homeostasis, handling oxidative stress and regulating metabolism. Downregulated pathways are related to gene regulation, particularly chromatin remodeling, RNA processing and protein transport (Fig. 6D). This suggests reduced gene expression control and protein modification after the treatment in female cells. Altogether, these data suggest that in male CP-epithelial cells, the sh-*angptl2* enhanced structural processes like cell morphogenesis and cell-to-cell contact formation, while decreasing functions in signal transduction and protein trafficking. In females, the sh-*angptl2* promoted metabolic regulation and stress response, and globally decreased the capacity for gene and protein processing.

### DEGs in OPCs

In the hippocampus, although they only represent 3% of the cells, OPCs play key roles in synaptic plasticity and neural communication, which are critical for learning and memory processes. In these cells, sh-*angptl2* down-regulated more DEGs than it up-regulated them, in males (481 down DEGs vs. 32 up DEGs) and in females (244 down DEGs vs. 70 up DEGs) (Fig. 6E). As in the other cell types, the pathways associated to those dysregulated genes differ between sexes and affect different biological processes. In male OPCs, up-regulated pathways are related to protein translation and transport, tube branching and formation, autophagy and iron-dependent metabolism, suggesting increase in protein turnover, cellular remodeling and morphogenesis. Down-regulated pathways focus on neuronal development, particularly axon guidance, neuron projection formation and synaptic transmission, suggesting reduced neural connectivity and plasticity (Fig. 6E). In female OPCs, up-regulated pathways are concentrated on neuronal signaling and synaptic transmission involving Wnt signaling and excitatory glutamate receptor pathways, suggesting enhanced neural communication and synaptic plasticity. Down-regulated pathways in female OPCs are all related to RNA processing and post-transcriptional regulation, indicating a reduction in gene expression control and RNA metabolic processes (Fig. 6E). Thus, after 3 months of sh-*angptl2*, and as observed in neurons and CP-epithelial cells, analysis of gene pathways suggests that male OPCs showed increased structural changes but reduced neuronal plasticity, while female OPCs exhibited enhanced neuronal signaling but decreased RNA processing.

### Cell-to-cell communications between CP-epithelial cells, OPCs and neurons

**Fig. 7:**
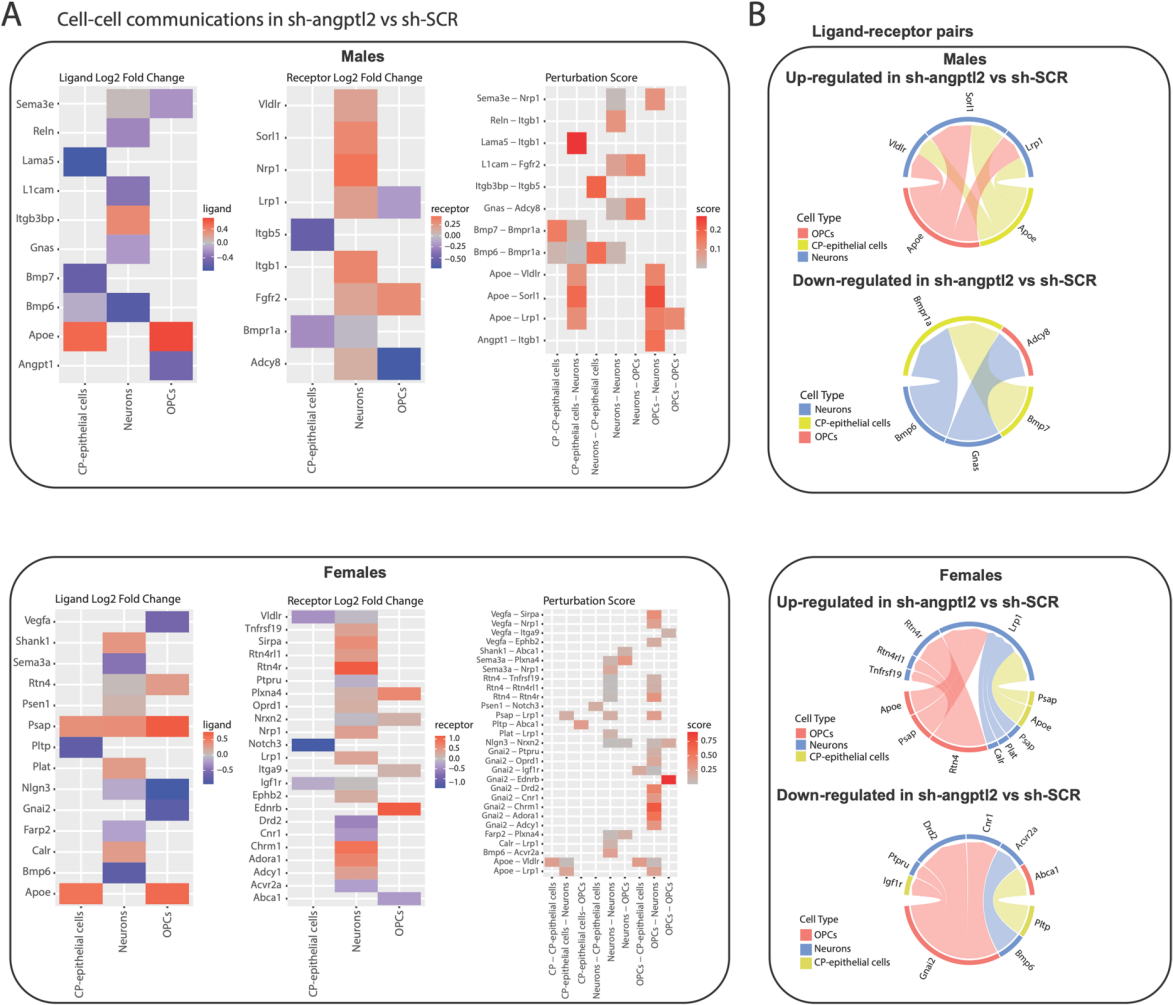
Cell–cell communication between neurons, OPCs and CP-epithelial cells in the brain of male and female ATX mice treated with sh-SCR or sh-*angptl2.* **A** Cell–cell communication analysis using the Connectome package, between OPCs, neuronal and CP-epithelial cells from male and female hippocampus, comparing the transcriptomic impact of sh-*angptl2* vs sh-SCR on ligands (left panels), receptors (middle panels) and ligand-receptors (right panels) perturbations. **B** Circos plots of most impacted ligand-receptor pairs by sh-*angptl2* in male and female neuronal-OPCs-CP-epithelial cells, with connecting lines representing significant communication links in the signaling network. The stringent significance criteria (log₂ fold change ≥ 0.25, gene expression present in at least 10% of cells *per* cell type, and *p* value < 0.05) were applied, and restricted to the three cell types identified by Augur

The divergent individual cell type changes in response to the treatment prompted us to consider cell-to-cell interactions to try to reconnect all individual changes. Using the Connectome R package, we calculated separately in each sex and in each cell-type the differential expression of ligands and receptors leading to a perturbation score of each ligand-receptor interaction, between sh-SCR and sh-*angptl2* groups (Fig. 7). We restricted our analysis to the three cell types identified by Augur to identify interactions biologically relevant, as they are specific to the most transcriptionally responsive cell populations. Furthermore, we applied stringent significance criteria—log₂ fold change ≥ 0.25, with gene expression present in at least 10% of cells *per* cell type, and *p* value < 0.05—which further narrowed the analysis to only the most robust ligand-receptor interactions. Interestingly, in male OPC and CP-epithelial cells, the only ligand upregulated by the treatment is *Apoe* (Fig. 7A), further highlighting its potential key role. To a lesser extent, *Itgb3bp* was also upregulated in male neurons, an integrin involved in cell migration and survival (Fig. 7A). The receptors *Vldlr*, *Sorl1*, *Nrp1* and *Lrp1* were up-regulated in male neurons (Fig. 7A–B); they contribute to lipid uptake and signaling pathways associated with synaptic plasticity and neuroprotection [33]. In addition, more than half of the ligand-receptor duos downregulated by the treatment in male cells concerned TGF-β family proteins (*Bmp6, Bmp7, Bmpr1a*) (Fig. 7B), which are involved in various aspects of development and differentiation, among others [34].

Female cells had more modified ligand-receptor duos than males (Fig. 7). The increase in ligands like *ApoE*, *Psap*, *Shank1*, *Rtn4*, *Calr* and *Plat* indicates a possible increase of signaling capabilities related to neuronal survival, inflammation and recovery processes [35]. Concerning the receptors, the up-regulation only in neurons of receptors like *Tnfrsf19*, *Rtn4rl1* and *Lrp1* may suggest enhanced signaling related to neuronal development and repair mechanisms, which is particularly important for maintaining synaptic health and facilitating recovery from injury [36]. Whereas neuroprotective ApoE was upregulated in both sexes, ApoE receptors were mostly expressed in males, supporting better synaptic resilience; in females, only *Lrp1* was slightly up-regulated in neurons, contrasting with the up-regulation of *Vldlr*, *Sorl1* and *Lrp1* in male neurons (Fig. 7A–B). In addition, the down-regulation of ligands like *Gnai2*, *BMP6* and *Pltp* in females may indicate a reduction in G protein signaling and BMP pathways (Fig. 7B), which could affect various cellular processes, including growth and differentiation [37]. The down-regulated receptors *Igf1r*, *Ptpru*, *Drd2*, *Cnr1*, *Acvr2a* and *Abca1* in females (Fig. 7B) suggest a decreased responsiveness to important growth factors and neurotransmitter signaling pathways, potentially impacting cellular communication and metabolic processes, and thus cognition [38, 39].

## Discussion

This study provides compelling evidence of sex-specific effects of sh-*angptl2* on cognition and cellular pathways in the hippocampal region of the brain of adult ATX mice. Our findings demonstrate that while delayed memory retention was restored in sh-*angptl2*-treated male mice, the treatment was deleterious in females on learning and short-term memory retention. Single-nucleus transcriptomic analysis of hippocampal cells revealed that senescent *angptl2*^+^ cells, the targets of the shRNA treatment, included CP-epithelial cells, neurons and OPCs. In contrast, hippocampal senescent EC expressed low levels of *angptl2* and were, therefore, not a major direct target of the active treatment; however, endothelial senescence pathways were downregulated by sh-*angptl2*, at least in male cells, suggesting that even a low expression of *angptl2* contributes to cellular senescence. Targeting *angptl2*^+^ senescent cells induced distinct cellular and molecular responses between males and females, influencing their respective cognitive outcomes. In neurons, OPC and CP-epithelial cells, the mitigated effects of sh-*angptl2* on DEGs and related pathways in male cells may explain the small, yet significant cognitive improvement observed in male mice; in contrast, the more global adverse molecular effects of sh-*angptl2* in female cells may explain the deleterious cognitive effect of the treatment in females. Our data are in support of recent studies, including ours in mice [15] and in patients with symptomatic coronary artery disease (CAD) [40], demonstrating a sexual dimorphism in response to various senotherapies favoring males [17, 18, 41]. Altogether, these data suggest that in male ATX mice, accumulation of non-vascular hippocampal senescent cells likely contributes to cognitive dysfunctions. In females, since targeting *angptl2*^+^ senescent cells is deleterious, the presence of senescent cells may be an efficient physiological mechanism of defense or repair, as recently suggested in patients with CAD [40].

The primary outcome of our study was cognition. We relied on the MWM test to assess the impact of the sh-*angptl2* treatment on cognitive functions such as learning and spatial memory. The active treatment, but not the sh-SCR, restored the delayed memory retention in male mice. In contrast, female mice, that had some delayed memory retention at 6 months old, lost this capacity at 9 months old, and the active treatment did not restore it. Importantly, the sh-*angptl2* treatment significantly reduced learning and suppressed short-term memory retention in female mice. This reveals a differential response to the sh-*angptl2* between sexes, aligning with prior evidence that cognitive aging and senotherapy efficacy vary by sex [15, 17, 18, 41]. Altogether, our data suggest that targeting *angptl2*^+^ senescent cells had a deleterious effect in female ATX mice and a beneficial effect in males.

To impact cognition, we originally chose to target senescent vascular cells, and in particular EC [23]. Based on multiple line of evidence, we indeed propose that ATX mice are a model of VCI: we reported that severe dyslipidemia impairs cerebral endothelial function [42, 43], and adversely affects the structure and biomechanical properties of cerebral arterial walls [43]. These changes could contribute to altered resting cerebral blood flow [43], impacting both the macro- and microvascular beds; this includes micro-bleedings and blood brain barrier leakage, along with cerebral hypoperfusion [14], ultimately leading to cognitive dysfunction [14, 42]. On the other hand, we reported that angptl2 induces atherosclerosis and is massively secreted by EC in ATX mice [22] and by senescent cells [21]. Our working hypothesis was therefore to test whether targeting vascular *angptl2*^+^ senescent cells would delay the cognitive decline associated with vascular dysfunctions in severely dyslipidemic ATX mice. In contrary to our hypothesis, the data of the present study suggest that targeting senescent *angptl2*^+^ cells did not improve any vascular component (ex vivo endothelial function and carotid compliance, in vivo blood flow index) in both sexes; the sh-*angptl2* modified cognitive functions by modulating the function of non-vascular cells (hippocampal neurons, CP-epithelial cells, OPCs) and, to a lower extent, by regulating senescence in EC, at least in male cells.

In both sexes, hippocampal senescent cells expressing *angptl2*, and thus targeted by the sh-*angptl2*, are *Glb1*^+^ neurons, *Glb1*^+^ CP-epithelial cells and *Glb1*^+^ OPCs. Senescent hippocampal *p21*^+^ EC express low transcript levels of *angptl2* and were, therefore, theoretically less sensitive to the treatment. Accordingly, sh-*angptl2* reduced *angptl2* transcript levels in neurons, CP-epithelial cells and to a lesser extent in OPCs, but not significantly in EC, at least in male mice. The fact that sh-*angptl2* targeted non-vascular cells in the hippocampus and did not lower *angptl2* transcript levels in EC was unexpected. Yet, levels of *angptl2* transcripts in EC were low, and thus differences may not have been readily detectable by snRNA-seq. In addition, the effects of the sh-*angptl2* are transient, and the analytic window chosen could have missed the changes of *angptl2* transcripts in the brain. Most importantly, targeting only *angptl2*^+^ cells did not eliminate all senescent cells, as *angptl2* alone is likely not solely responsible for driving senescence in the brain. Thus, senescent hippocampal ECs that do not express *angptl2* were not a primary target of the treatment. This may explain, at least partially, the absence of beneficial effects of sh-*angptl2* on vascular functions. Nevertheless, senescence gene-related pathways were downregulated in all cell types, including in EC, in sh-*angptl2* male ATX mice; in females, cellular senescence was upregulated, in all cell types, particularly in female EC. This suggests that senescent EC are somehow responsive to the sh-*angptl2*, raising the possibility that vascular senescent *angptl2*-negative cells may contribute to age-related cognitive impairments in ATX mice. We previously reported that targeting *angptl2* is senolytic in male mice, inducing senescent cell death by apoptosis [23]. Accordingly, in the present study sh-*angptl2* also killed senescent neurons, CP-epithelial cells and OPCs, but also EC (in which *p21* expression was decreased by the treatment) by promoting their apoptosis in male cells. In female ATX mice, the sh-*angptl2* promoted senescence, despite a small pro-apoptotic effect. Altogether, this suggests that senescent *angptl2*^+^ vascular cells specifically—but not senescent vascular cells in general—do not play a key role in VCI. In contrast, non-vascular *angptl2*^+^ senescent cells may contribute to cognitive decline in ATX mice, particularly in males.

To the best of our knowledge, our study is the first to look simultaneously at the impact of a senolytic treatment on cognitive functions and single-cell RNA sequencing in the brain. Indeed, although it had been shown that targeting senescence improved cognitive functions in aging mice [11], or in mice exposed to traumatic brain injury [18], for example, scRNA-seq performed in these studies did not assess the effect of the senolytics in treated brain cells. Instead, these analyses were performed to specifically evaluate the effects of aging or trauma, not the impact of a senolytic treatment. The exact cell types and molecular pathways targeted by senolysis remain to be determined. Our study therefore adds to the field by linking cognitive dysfunction with molecular pathways in brain cells in a murine model of CVD.

Because cognition was modulated by the senotherapy, we expected to detect changes in molecular pathways in neurons. In male neurons, we observed a limited up-regulation of mitochondrial and energy metabolism pathways, which was paralleled by the repression of numerous genes involved in synaptic transmission. It is known that improved mitochondrial efficiency can promote cognition by providing the energy required for synaptic remodeling and neuronal signaling [44]. But the massive down-regulated genes and related pathways suggest rather a reduction in synaptic transmission and neuronal plasticity. Alternatively, to explain the observed memory benefits in male mice, synaptic remodeling, and more specifically synaptic pruning (natural process where the brain eliminates extra synapses), may promote cognitive adaptability, potentially clearing weaker synapses and reinforcing stronger neuronal circuits [45]. In contrast, female neurons responded to the treatment with up-regulation of neuron development pathways and down-regulation of GABAergic signaling. These findings suggest that the treatment disrupted the balance between excitatory and inhibitory signals, which might have impaired female mice cognitive functions such as learning and memory. GABAergic signaling plays a crucial role in maintaining inhibitory control in the hippocampus, and alterations in this pathway have been linked to cognitive deficits [46]. Because cognition was impaired in females, it is likely that proper cell-to-cell connections are inadequate and that the neuronal molecular remodeling is maladaptive.

The differential response of OPCs also revealed interesting sex-specific patterns. In males, down-regulation of OPCs-related synaptic and neuronal development pathways suggests, like in neurons, a reduction in cellular plasticity following treatment. This could indicate a shift toward maintenance rather than development of new cellular connections, aligning with up-regulation of cellular remodeling and the observed memory restoration. In contrast, female OPCs exhibited up-regulation of Wnt signaling pathways known to drive neurogenesis and synaptic plasticity [47] that would support neuronal developmental pathways. Despite this up-regulation, however, down-regulation of RNA processing pathways in females could prevent efficient gene expression, in turn limiting the capacity for neurogenesis and repair. Hence, while females may attempt to promote synaptic plasticity in response to the treatment with sh-*angptl2*, they may not be able to execute these processes efficiently due to impaired RNA processing, a defect known to alter neuronal development [48]. Thus, in females both neurons and OPC appear to simultaneously engage an adaptive remodeling in response to the senolytic treatment that is, ultimately, inadequate.

Based on Augur analysis, the most affected cell type by the treatment in a sex-specific manner was the CP-epithelial cells, where male cells showed increased synaptic formation and reduced intracellular signaling and cytoskeletal organization, indicating a potential decrease in cell motility and structural changes. In contrast, female cells exhibited an up-regulation of stress response pathways, suggesting that the sh-*angptl2* might cause cellular stress in females more than in males. Oxidative stress and cellular damage are closely linked to aging and neurodegeneration [49]. Therefore, in the female brain, neurons and OPC respond to the treatment by a maladaptive molecular remodeling while CP-epithelial cells express stress response pathways; taken together, this might suggest that the treatment induced stress rather than benefit in the brain of female ATX mice, leading to deleterious effects on cognitive functions.

Altogether, these data suggest a coordinated response to the senolytic treatment of neurons, CP-epithelial cells and OPCs, at least in male cells. We therefore focused our analyses on receptor/ligand interactomes that may support collaborative cell–cell adaptations to the senotherapy, in these three cell types. Among all the ligand-receptor duos studied, only *ApoE*, a gene implicated in lipid metabolism and neuronal repair, was up-regulated in both sexes, in CP-epithelial cells and in OPCs. Increased *ApoE* expression may reflect an attempt to maintain neuronal homeostasis and repair damaged cells [50]. However, the more favorable outcomes in male mice may be attributed to the parallel up-regulation of the associated neuroprotective receptors for ApoE such as *Vldlr*, *Lrp1* and *Sorl1*, known to enhance synaptic plasticity and prevent neurodegeneration [51]. The absence of these receptor pathways in females suggests a less robust neuroprotective response, possibly contributing to the sex-specific differences in learning and retention memory observed in the MWM test.

### Limitations of the study

- The large number of variables—namely sex, treatment and the diverse hippocampal cell types—limits the depth of data interpretation. Given the thousands of DEGs and molecular changes induced by the senolytic treatment, we have only begun to uncover the complex, interdependent positive and negative variations. Any associations between the transcriptomic signatures and cognitive functions remain speculative and are based solely on bioinformatic analyses.
- Another limitation is that targeting angptl2⁺ senescent cells had little to no detectable impact on EC function in pial and microvascular arteries; similarly, the treatment had a lower effect on the hippocampal EC transcriptomic profile compared to its impact on non-vascular cell types. Angptl2 being secreted by senescent ECs [21], we expected that sh-*angptl2* would have significantly decreased *angptl2* expression in ECs, improved endothelial-dependent functions and enriched endothelial-DEGs related pathways to ameliorate neurovascular functions, and ultimately, cognition. Although sh-*angptl2* treatment improved cognition—at least in male mice—and EC senescence pathways were more pronounced and reversed by sh-*angptl2* in males, senescent neurons, OPCs and CP-epithelial cells emerged as key targets of sh-*angptl2*. These findings suggest that non-vascular senescent angptl2⁺cells, rather than vascular senescent angptl2⁺cells, are the main contributor to cognitive deficits in ATX mice, contrary to our original hypothesis. More experiments will also be needed to understand why low expression of *angptl2* is associated with a high expression of *Cdkn1a* in EC, while high expression of *angptl2* is associated with a high expression of *Glb1* transcripts in the other cell types.
- Since targeting *angptl2*+ senescent cells in female mice had deleterious consequences for cognitive functions, this may suggest that in female ATX mice, senescence could be an efficient defense/repair mechanism to regulate hippocampal functions. This remains to be elucidated.
- We do not know specifically whether (i) the treatment has a direct effect on each cell type, or ii) if the response to the sh-*angptl2* on one cell type is sufficient to trigger changes in the other cells families. For example, the impact of sh-*angptl2* on senescent EC, which express very low levels of *angptl2*, may be indirect and potentially mediated through paracrine signaling from sh-*angptl2*-sensitive cells.
- Our choice of treatment design, with two injections of AAV1-shRNA over 3 months, targeting the single gene *angptl2*, unlike using a pan-senolytic therapy, may be considered as a limitation. In addition, temporary and modest reduction of *angptl2* expression levels could be considered a limitation, suggesting potential variability in viral vector efficiency or durability of the shRNA effect. More injections, however, would likely have stimulated the expression of antibodies against AAV1 [52]. Globally, senesence can be targeted by senolytics drugs, such as dasatinib/quercetin, fisetin, navitoclax, quercetin, by senomorphics that will target SASP factors such as NFkB inhibitors, or more rarely by gene therapy, targeting directly p16, for example [53]. However, similar to our approach using sh-*angptl2*, senolytic treatments are typically administrated in short cycles to minimize toxicity, commonly involving 1 week of treatment followed by a 2–3 week recovery period, repeated over approximately 3 months.
- The parallel use of wild-type mice would better contextualize the extent of the atherosclerotic disease and permit to determine whether sh-*angptl2* treatment leads to normalization toward a healthy phenotype in ATX mice, at least in male mice. In this study, however, this would have complexified the analysis even further.

Therefore, multiple questions remain unanswered.

In conclusion, our data support the concept that accumulation of non-vascular *angptl2*^+^ senescent cells contributes to cognitive impairment in male ATX mice, but not in females. Our results highlight the importance of considering sex as a critical biological variable in therapeutic development, particularly in the context of senolytics, in light of recent literature. While targeting senescence with sh-*angptl2* appears to offer some cognitive benefits in males, like with other senolytics tested so far, senolytic treatment may not be as effective (or might even be detrimental) in females. Understanding the interactions between sex, aging, dyslipidemia and interconnected cellular pathways will be critical in developing effective interventions for age-related cognitive decline and neurodegenerative diseases associated with CVD.

## Supplementary Information

Below is the link to the electronic supplementary material.

Supplementary file1 (DOCX 8289 KB)

## Data Availability

Data will be made available upon reasonable request.

## Declarations

### Data sharing

All single-nuclei data for this study have been deposited in the US National Center for Biotechnology Information gene Expression Omnibus database under the number GSE278331.

## References

[1] Childs BG, et al. Senescent cells: an emerging target for diseases of ageing. Nat Rev Drug Discov. 2017;16(10):718–35. 28729727 10.1038/nrd.2017.116 PMC5942225

[2] Coppé JP, et al. The senescence-associated secretory phenotype: the dark side of tumor suppression. Annu Rev Pathol. 2010;5:99–118. 20078217 10.1146/annurev-pathol-121808-102144 PMC4166495

[3] Liberale L, et al. Inflammation, aging, and cardiovascular disease: JACC review topic of the week. J Am Coll Cardiol. 2022;79(8):837–47. 35210039 10.1016/j.jacc.2021.12.017 PMC8881676

[4] Kiss T, et al. Single-cell RNA sequencing identifies senescent cerebromicrovascular endothelial cells in the aged mouse brain. Geroscience. 2020;42(2):429–44. 32236824 10.1007/s11357-020-00177-1 PMC7205992

[5] Kiss T, et al. Spatial transcriptomic analysis reveals inflammatory foci defined by senescent cells in the white matter, hippocampi and cortical grey matter in the aged mouse brain. Geroscience. 2022;44(2):661–81. 35098444 10.1007/s11357-022-00521-7 PMC9135953

[6] Shafqat A, et al. Cellular senescence in brain aging and cognitive decline. Front Aging Neurosci. 2023;15:1281581. 38076538 10.3389/fnagi.2023.1281581 PMC10702235

[7] Bussian TJ, et al. Clearance of senescent glial cells prevents tau-dependent pathology and cognitive decline. Nature. 2018;562(7728):578–82. 30232451 10.1038/s41586-018-0543-y PMC6206507

[8] Musi N, et al. Tau protein aggregation is associated with cellular senescence in the brain. Aging Cell. 2018;17(6):e12840. 30126037 10.1111/acel.12840 PMC6260915

[9] Fatt MP, et al. Restoration of hippocampal neural precursor function by ablation of senescent cells in the aging stem cell niche. Stem Cell Reports. 2022;17(2):259–75. 35063124 10.1016/j.stemcr.2021.12.010 PMC8828532

[10] Tarantini S, et al. Treatment with the BCL-2/BCL-xL inhibitor senolytic drug ABT263/Navitoclax improves functional hyperemia in aged mice. Geroscience. 2021;43(5):2427–40. 34427858 10.1007/s11357-021-00440-z PMC8599595

[11] Zhang X, et al. Rejuvenation of the aged brain immune cell landscape in mice through p16-positive senescent cell clearance. Nat Commun. 2022;13(1):5671. 36167854 10.1038/s41467-022-33226-8 PMC9515187

[12] Faakye J, et al. Preventing spontaneous cerebral microhemorrhages in aging mice: a novel approach targeting cellular senescence with ABT263/navitoclax. Geroscience. 2024;46(1):21–37. 38044400 10.1007/s11357-023-01024-9 PMC10828142

[13] Toth P, et al. Functional vascular contributions to cognitive impairment and dementia: mechanisms and consequences of cerebral autoregulatory dysfunction, endothelial impairment, and neurovascular uncoupling in aging. Am J Physiol Heart Circ Physiol. 2017;312(1):H1–20. 27793855 10.1152/ajpheart.00581.2016 PMC5283909

[14] de Montgolfier O, et al. Systolic hypertension-induced neurovascular unit disruption magnifies vascular cognitive impairment in middle-age atherosclerotic LDLr(-/-):hApoB(+/+) mice. Geroscience. 2019;41(5):511–32. 31093829 10.1007/s11357-019-00070-6 PMC6885084

[15] Lambert M, et al. The senolytic ABT-263 improves cognitive functions in middle-aged male, but not female, atherosclerotic LDLr(-/-);hApoB(100)(+/+) mice. Geroscience. 2025;47(3):4577–600. 10.1007/s11357-025-01563-3. 39982668 10.1007/s11357-025-01563-3 PMC12181456

[16] Behfar Q, Zuniga AR, Martino-Adami PV. Aging, senescence, and dementia. J Prev Alzheimers Dis. 2022;9(3):523–31. 35841253 10.14283/jpad.2022.42

[17] Fang Y, et al. Sexual dimorphic metabolic and cognitive responses of C57BL/6 mice to Fisetin or Dasatinib and quercetin cocktail oral treatment. Geroscience. 2023;45(5):2835–50. 37296266 10.1007/s11357-023-00843-0 PMC10643448

[18] Schwab N, et al. Neurons and glial cells acquire a senescent signature after repeated mild traumatic brain injury in a sex-dependent manner. Front Neurosci. 2022;16:1027116. 36408415 10.3389/fnins.2022.1027116 PMC9669743

[19] Labbe P, et al. Angiopoietin-like 2 is essential to aortic valve development in mice. Commun Biol. 2022;5(1):1277. 36414704 10.1038/s42003-022-04243-6 PMC9681843

[20] Thorin-Trescases N, Thorin E. Angiopoietin-like-2: a multifaceted protein with physiological and pathophysiological properties. Expert Rev Mol Med. 2014;16:e17. 25417860 10.1017/erm.2014.19

[21] Thorin-Trescases N, et al. Angptl2 is a marker of cellular senescence: the physiological and pathophysiological impact of Angptl2-related senescence. Int J Mol Sci. 2021;22(22):12232. 10.3390/ijms222212232. 34830112 10.3390/ijms222212232 PMC8624568

[22] Farhat N, et al. Angiopoietin-like 2 promotes atherogenesis in mice. J Am Heart Assoc. 2013;2(3):e000201. 23666461 10.1161/JAHA.113.000201 PMC3698785

[23] Caland L, et al. Knockdown of angiopoietin-like 2 induces clearance of vascular endothelial senescent cells by apoptosis, promotes endothelial repair and slows atherogenesis in mice. Aging (Albany NY). 2019;11(11):3832–50. 31186381 10.18632/aging.102020 PMC6594793

[24] Amadatsu T, et al. Macrophage-derived angiopoietin-like protein 2 exacerbates brain damage by accelerating acute inflammation after ischemia-reperfusion. PLoS ONE. 2016;11(11):e0166285. 27861531 10.1371/journal.pone.0166285 PMC5115716

[25] Hiramoto K, et al. Decreased memory and learning ability mediated by Bmal1/M1 macrophages/angptl2/inflammatory cytokine pathway in mice exposed to long-term blue light irradiation. Curr Issues Mol Biol. 2024;46(5):4924–34. 38785563 10.3390/cimb46050295 PMC11120424

[26] Huang X, et al. ANGPTL2 deletion attenuates neuroinflammation and cognitive dysfunction induced by isoflurane in aged mice through modulating MAPK pathway. Mediators Inflamm. 2023;2023:2453402. 36865085 10.1155/2023/2453402 PMC9974309

[27] Baker DJ, Petersen RC. Cellular senescence in brain aging and neurodegenerative diseases: evidence and perspectives. J Clin Invest. 2018;128(4):1208–16. 29457783 10.1172/JCI95145 PMC5873891

[28] Ku T, Choi C. Noninvasive optical measurement of cerebral blood flow in mice using molecular dynamics analysis of indocyanine green. PLoS ONE. 2012;7(10):e48383. 23119000 10.1371/journal.pone.0048383 PMC3485229

[29] Bolduc V, et al. Heart rate-associated mechanical stress impairs carotid but not cerebral artery compliance in dyslipidemic atherosclerotic mice. Am J Physiol Heart Circ Physiol. 2011;301(5):H2081–92. 21926346 10.1152/ajpheart.00706.2011 PMC3700878

[30] Mury P, et al. Senescence and inflamm-aging are associated with endothelial dysfunction in men but not women with atherosclerosis. JACC Basic Transl Sci. 2024;9(10):1163–77. 39534645 10.1016/j.jacbts.2024.06.012 PMC11551873

[31] Liu G, et al. Measurements of cerebrospinal fluid production: a review of the limitations and advantages of current methodologies. Fluids Barriers CNS. 2022;19(1):101. 36522656 10.1186/s12987-022-00382-4 PMC9753305

[32] Flowers SA, Rebeck GW. APOE in the normal brain. Neurobiol Dis. 2020;136:104724. 31911114 10.1016/j.nbd.2019.104724 PMC7002287

[33] Eggert S, et al. Trafficking in Alzheimer’s disease: modulation of APP transport and processing by the transmembrane proteins LRP1, SorLA, SorCS1c, sortilin, and calsyntenin. Mol Neurobiol. 2018;55(7):5809–29. 29079999 10.1007/s12035-017-0806-x

[34] Krieglstein K, et al. More than being protective: functional roles for TGF-beta/activin signaling pathways at central synapses. Trends Neurosci. 2011;34(8):421–9. 21742388 10.1016/j.tins.2011.06.002

[35] Parhizkar S, Holtzman DM. APOE mediated neuroinflammation and neurodegeneration in Alzheimer’s disease. Semin Immunol. 2022;59:101594. 35232622 10.1016/j.smim.2022.101594 PMC9411266

[36] Faissner A. Low-density lipoprotein receptor-related protein-1 (LRP1) in the glial lineage modulates neuronal excitability. Front Netw Physiol. 2023;3:1190240. 37383546 10.3389/fnetp.2023.1190240 PMC10293750

[37] Farsi Z, et al. Brain-region-specific changes in neurons and glia and dysregulation of dopamine signaling in Grin2a mutant mice. Neuron. 2023;111(21):3378–96. 37657442 10.1016/j.neuron.2023.08.004

[38] Dyer AH, et al. The role of Insulin-Like Growth Factor 1 (IGF-1) in brain development, maturation and neuroplasticity. Neuroscience. 2016;325:89–99. 27038749 10.1016/j.neuroscience.2016.03.056

[39] Jacobs FM, et al. Identification of Dlk1, Ptpru and Klhl1 as novel Nurr1 target genes in meso-diencephalic dopamine neurons. Development. 2009;136(14):2363–73. 19515692 10.1242/dev.037556 PMC3266485

[40] Mury P, et al. Quercetin reduces vascular senescence and inflammation in symptomatic male but not female coronary artery disease patients. Aging Cell. 2025;15:e70108. 10.1111/acel.70108. 10.1111/acel.70108 PMC12341813 40375481

[41] Rani A, et al. Failure of senolytic treatment to prevent cognitive decline in a female rodent model of aging. Front Aging Neurosci. 2024;16:1384554. 38813533 10.3389/fnagi.2024.1384554 PMC11133672

[42] Drouin A, et al. Catechin treatment improves cerebrovascular flow-mediated dilation and learning abilities in atherosclerotic mice. Am J Physiol Heart Circ Physiol. 2011;300(3):H1032–43. 21186270 10.1152/ajpheart.00410.2010 PMC3702511

[43] Bolduc V, et al. Catechin prevents severe dyslipidemia-associated changes in wall biomechanics of cerebral arteries in LDLr-/-:hApoB+/+ mice and improves cerebral blood flow. Am J Physiol Heart Circ Physiol. 2012;302(6):H1330–9. 22268108 10.1152/ajpheart.01044.2011 PMC3695886

[44] Song N, et al. Focusing on mitochondria in the brain: from biology to therapeutics. Transl Neurodegener. 2024;13(1):23. 38632601 10.1186/s40035-024-00409-w PMC11022390

[45] Cornell J, et al. Microglia regulation of synaptic plasticity and learning and memory. Neural Regen Res. 2022;17(4):705–16. 34472455 10.4103/1673-5374.322423 PMC8530121

[46] Jimenez-Balado J, Eich TS. GABAergic dysfunction, neural network hyperactivity and memory impairments in human aging and Alzheimer’s disease. Semin Cell Dev Biol. 2021;116:146–59. 33573856 10.1016/j.semcdb.2021.01.005 PMC8292162

[47] Narvaes RF, Furini CRG. Role of Wnt signaling in synaptic plasticity and memory. Neurobiol Learn Mem. 2022;187:107558. 34808336 10.1016/j.nlm.2021.107558

[48] Fliedner A, et al. Variants in SCAF4 cause a neurodevelopmental disorder and are associated with impaired mRNA processing. Am J Hum Genet. 2020;107(3):544–54. 32730804 10.1016/j.ajhg.2020.06.019 PMC7477272

[49] Olufunmilayo EO, Gerke-Duncan MB, Holsinger RMD. Oxidative stress and antioxidants in neurodegenerative disorders. Antioxidants (Basel). 2023;12(2):517. 10.3390/antiox12020517. 36830075 10.3390/antiox12020517 PMC9952099

[50] Fernandez-Calle R, et al. APOE in the bullseye of neurodegenerative diseases: impact of the APOE genotype in Alzheimer’s disease pathology and brain diseases. Mol Neurodegener. 2022;17(1):62. 36153580 10.1186/s13024-022-00566-4 PMC9509584

[51] May P, et al. Neuronal LRP1 functionally associates with postsynaptic proteins and is required for normal motor function in mice. Mol Cell Biol. 2004;24(20):8872–83. 15456862 10.1128/MCB.24.20.8872-8883.2004 PMC517900

[52] Ertl HCJ. Immunogenicity and toxicity of AAV gene therapy. Front Immunol. 2022;13:975803. 36032092 10.3389/fimmu.2022.975803 PMC9411526

[53] Zheng L, et al. Targeting cellular senescence in aging and age-related diseases: challenges, considerations, and the emerging role of senolytic and senomorphic therapies. Aging Dis. 2024;15(6):2554–94. 38421832 10.14336/AD.2024.0206 PMC11567261

