# Supplementary material for: Targeting angiopoietin like-2 positive senescent cells improves cognitive impairment in adult male but not female atherosclerotic LDLr−/−;hApoB100+/+ mice
Source: GeroScience. 2025 Jun 30;47(6):6999–7022. doi: 10.1007/s11357-025-01763-x (PMC12638535; doi:10.1007/s11357-025-01763-x)
Supplement: Supplementary file 1 — Supplementary file1 (DOCX 8289 KB) [file 11357_2025_1763_MOESM1_ESM.docx]

**Targeting angiopoietin like-2 positive senescent cells improves cognitive impairment in adult male but not female atherosclerotic LDLr^-/-^;hApoB_100_^+/+^** **mice**

Mélanie Lambert^1,2^, Géraldine Miquel^2^, Gael Cagnone^3^, Pauline Mury^1,2^, Louis Villeneuve^2^, Frédéric Lesage^4^, Nathalie Thorin-Trescases^2^, Eric Thorin^2,5^

**Supplemental data**

**Fig. S1:** Impact of sh-*angptl2* on body weight over the 3 months of treatment.

**Fig. S2:** Cognition in the MWM test at 6-month of age, before the treatment was started.

**Fig. S3:** Impact of sh-*angptl2* treatment on atherosclerotic lesion, blood flow index, carotid compliance and cerebral endothelial dilatory function.

**Fig. S4:** Impact of sh-*angptl2* treatment on cognition in the MWM test in mice selected for single-nuclei RNA sequencing analysis.

**Table S1**: Sequence of sh-*angptl2* or sh-SCR

**Table S2**: Sequence of primers for AAV1-pU6-ITR-sh (BGH-poly A) titration

**Table S3**: List of primers used for real-time quantitative PCR

**Table S4**: Impact of sh-*angptl2* or sh-SCR treatment on blood markers

**Table S5**: Proportion (%) of the identified cell types in male and female hippocampal region

**
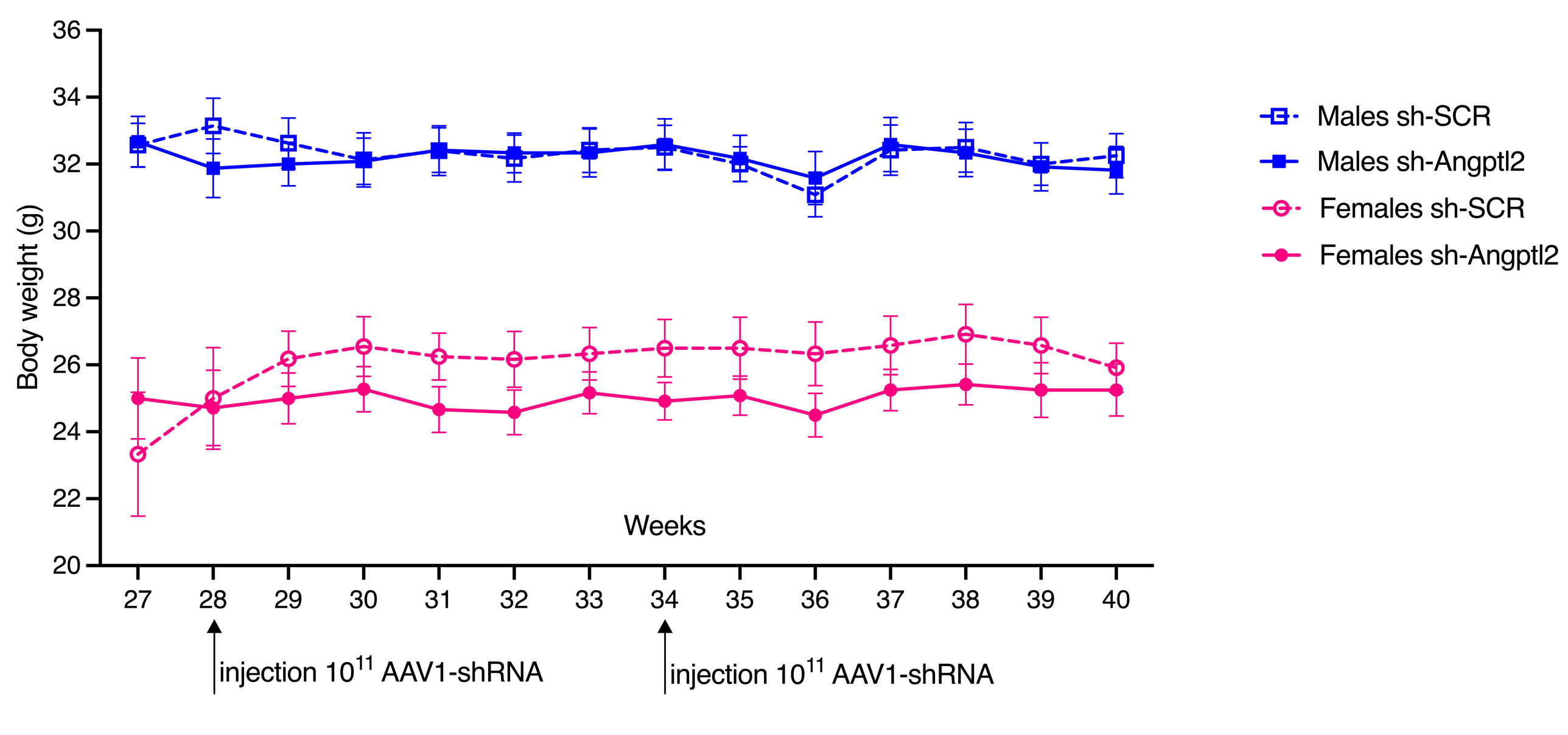
**

**Figure Supplemental 1: Impact of sh-*angptl2* on body weight over the 3 months of treatment.** Data are expressed as mean±SEM of n mice (n=12 male sh-SCR, n=12 male sh-*angptl2*, n=13 female sh-SCR and n=11 female sh-*angptl2* mice).

**
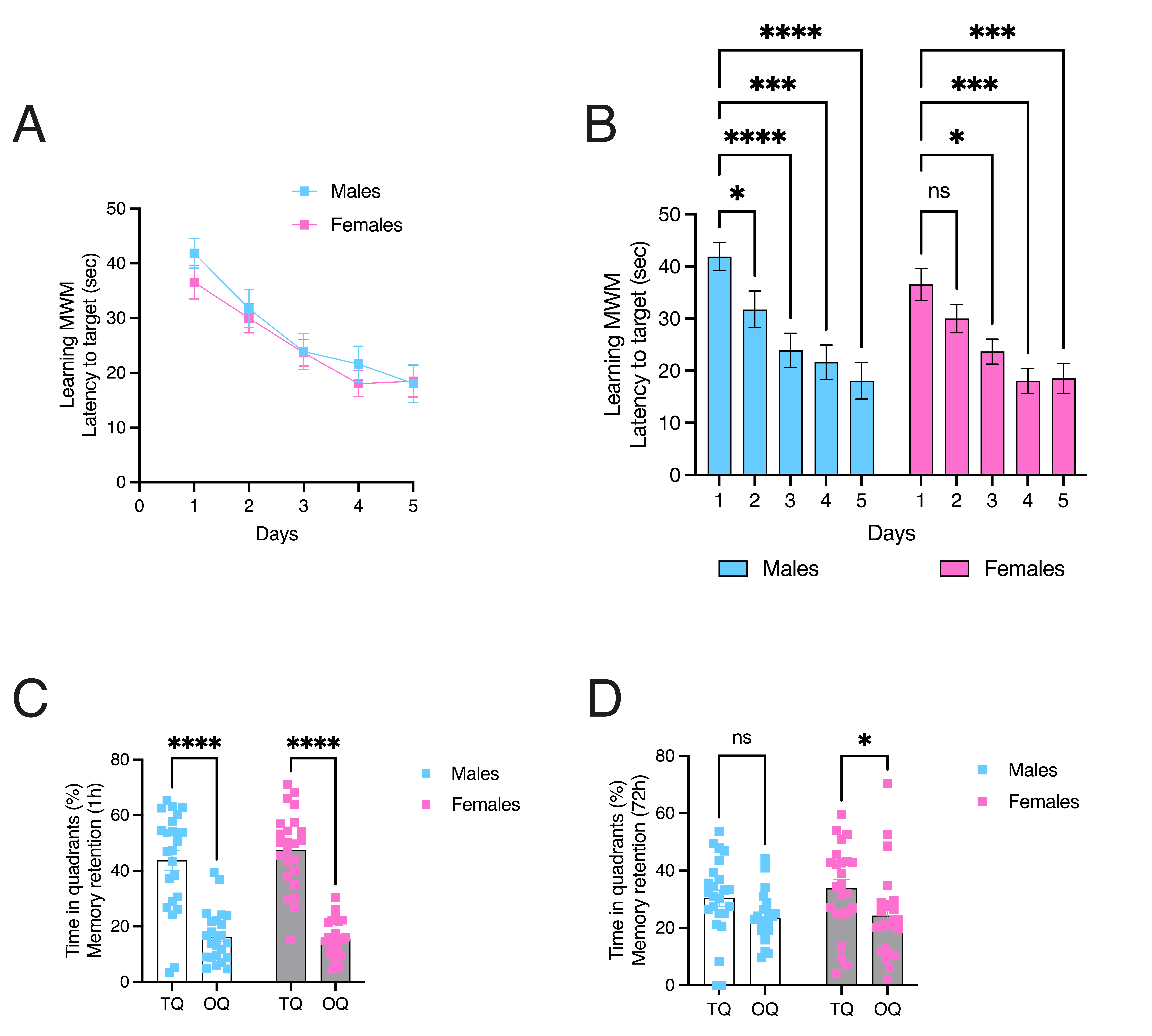
**

**Figure Supplemental 2: Cognition in the MWM** **test at 6-month of age, before the treatment was started** (**A-B**) Latency to find the hidden platform from day 1 to day 5 in the acquisition phase (learning memory) of the Morris water maze (MWM) test of male and female ATX mice. Each value is a mean of 4 trials performed by each mouse, per day of learning. Latency to target was also analyzed to determine in each group at what day the mice started to remember the localization of the hidden platform in male and female mice: * p<0.05 *vs*. Day1, Two-way ANOVA with repeated measures (Days x Treatment) and Dunnet’s multiple comparisons test. (**C-D**) Percentage of time spent in the target quadrant (TQ, quadrant in which the platform was hidden) and the opposite quadrant (OQ) by male and female mice during the probe test. This probe test was performed on day 5, 1h after the last acquisition of the learning phase (short-term memory retention, **C**) and 72h after the end of the learning phase (delayed memory retention, **D**). *: p<0.05 *vs.* TQ, Two-way ANOVA (Sex x Treatment) and Sidak’s multiple comparisons test. N=24 male and n=24 female mice. Data are expressed as mean±SEM of n mice. *: p<0.05; ****: p<0.0001.

**

**

**Figure Supplemental 3: Impact of sh-*angptl2* treatment on cognition in the MWM** **test in mice selected for single-nuclei RNA sequencing analysis.** Percentage of time spent in the target quadrant (TQ, quadrant in which the platform was hidden) and the opposite quadrant (OQ) by male and female mice after treatment with sh-SCR or sh-*angptl2* during the probe tests. These probe tests were performed on day 5, 1h after the last acquisition of the learning phase (short-term memory retention, **A**) and 72h after the end of the learning phase (delayed memory retention, **B**). *: p<0.05 *vs.* TQ. Two-way ANOVA (Sex x Treatment) and Sidak’s multiple comparisons test. N=6 mice at 6-month of age and n=3 at 9-month of age, per group. Data are expressed as mean±SEM of n mice. *: p<0.05; **: p<0.01; ***: p<0.001.

**Table S1**: Sequence of sh-*angptl2* or sh-SCR

| sh-*angptl2* | GCAGAGTCTTCCAATCAGTTAATCAAGAGTTAACTGATTGGAAGACTCTGC |
| --- | --- |
| sh-SCR | CCTAAGGTTAAGTCGCCCTCGCTCGAGCGAGGGCGACTTAACCTTAGG |

**Table S2** : Sequence of primers for AAV1-pU6-ITR-sh (BGH-poly A ) titration

| BGH-Forward | 5’TGCCTTCCTTGACCCT |
| --- | --- |
| BGH-Reverse | 5’CCTTGCTGTCCTGCCC |

**Table S3**: List of primers used for real-time quantitative PCR

| **Target gene** | **Primers** | **Sequences** |
| --- | --- | --- |
| *Angptl2* | Forward | GATCCAGAGTGACCAGAATC |
|  | Reverse | TCTCAGGCTTCACCAGGTAG |
| *CycloA* | Forward | CCGATGACGAGCCCTTGG |
|  | Reverse | GCCGCCAGTGCCATTATG |
| *HPRT* | Forward | GGTTAAGCAGTACAGCCCCA |
|  | Reverse | GGCCTGTATCCAACACTTCG |
| *BM2* | Forward | TCTCACTGACCGGCCTGTAT |
|  | Reverse | GATTTCAATGTGAGGCGGGTG |
| *PPIA* | Forward | CCACCGTGTTCTTCGACATC |
|  | Reverse | CTGGCACATGAATCCTGGAA |

**Table S4**: Impact of sh-*angptl2* or sh-SCR treatment on blood markers

|  | **Males-**  **sh-SCR** | **Males-**  **sh-*angptl2*** | **Females-**  **sh-SCR** | **Females-**  **sh-*angptl2*** |
| --- | --- | --- | --- | --- |
| Glucose (mmol/L) | 12.7±1.3 (8) | 13.4±1.1 (6) | 11.9±1.0 (8) | 10.9±0.6 (6) |
| Cholesterol (mmol/L) | 27.6±1.0 (8) | 26.0±1.6 (7) | 27.2±1.4 (8) | 30.7±0.9 (7) |
| Triglycerides (mmol/L) | 8.6±0.5 (8) | 7.5±0.8 (7) | 5.4±0.3 (8) **#** | 5.4±0.3 (7) |
| Urea (mmol/L) | 9.0±1.1 (8) | 6.3±0.3 (7) | 7.5±0.4 (8) | 6.7±0.6 (7) |
| AST (U/L) | 269.4±63.2 (8) | 340.8±163.3 (6) | 176.9±14.3 (8) | 207.9±22.1 (7) |
| Phosphatase alkaline (U/L) | 70.0±13.0 (5) | 58.8±1.3 (4) | 91.9±7.1 (8) | 95.7.7±4.6 (7) |

Data are mean ± SEM of (n) mice; 2-way ANOVA, (Sex x Treatment) and Tukey’s multiple comparisons test. #: p<0.05 *vs*. Males (within the same treatment).

**Table S5**: Proportion (%) of the identified cell types in male and female hippocampal region

| % | **Male**  **sh-SCR** | **Male**  **sh-*angptl2*** | **Female**  **sh-SCR** | **Female**  **sh-*angptl2*** |
| --- | --- | --- | --- | --- |
| Neurons | 58.12 | 74.35 | 54.06 | 56.95 |
| Oligodendrocytes | 21.98 | 10.07 | 24.73 | 23.22 |
| Astrocytes | 11.3 | 8.38 | 12.70 | 10.76 |
| OPCs | 3.16 | 2.77 | 3.13 | 2.48 |
| ECs | 2.16 | 1.39 | 1.73 | 2.75 |
| Immune cells | 1.78 | 0.96 | 1.95 | 2.41 |
| Epithelial cells | 1.49 | 2.10 | 1.71 | 1.43 |

Data are average proportion of cells from n=3 mice (hippocampi) *per* group.
